# Transitive interactions among rhizobia determine their symbiotic fitness

**DOI:** 10.1093/ismeco/ycag210

**Published:** 2026-07-21

**Authors:** Margarita Granada Agudelo, Bryan Ruiz, Jean-Baptiste Ferdy, Delphine Capela, Philippe Remigi

**Affiliations:** Univ Toulouse, CNRS, INRAE, LIPME, Castanet-Tolosan, France; Univ Toulouse, CNRS, INRAE, LIPME, Castanet-Tolosan, France; Univ Toulouse, Toulouse INP, CNRS, IRD, CRBE, Toulouse, France; Univ Toulouse, CNRS, INRAE, LIPME, Castanet-Tolosan, France; Univ Toulouse, CNRS, INRAE, LIPME, Castanet-Tolosan, France

**Keywords:** legume–rhizobia symbiosis, mutualism, microbe–microbe interactions, host control mechanisms, symbiont fitness, transitive interactions

## Abstract

During host–microbe symbioses, the fitness of mutualistic microbes is determined by the interactions that concurrently occur, throughout their life cycle, with their host and other members of the surrounding microbial community. Disentangling how these multiple interactions shape the fitness of microbial symbionts is challenging but is essential to understand the diversity and functioning of mutualisms. Here, we examined the different fitness components of rhizobial symbionts of the legume plant *Mimosa pudica* across the multiple stages of their symbiotic life cycle. By comparing rhizobial symbiotic fitness in single and pairwise inoculations, we found that interbacterial interactions causing significant fitness effects are common and can have major consequences, sometimes leading to the extinction of a strain. These interactions predominantly occur at the root infection (nodulation) step, but weaker postinfection interaction effects, involving yet uncharacterized mechanisms, were also detected. Furthermore, pairwise interaction effects were transitive and enabled to predict fitness ranks in more complex rhizobial communities consisting of six or eight strains, indicating that higher-order interaction effects do not play a significant role in these communities. Overall, our results provide a quantitative framework to describe the main drivers of rhizobial symbiotic fitness in a simple community context.

## Graphical Abstract

**Graphical Abstract:**
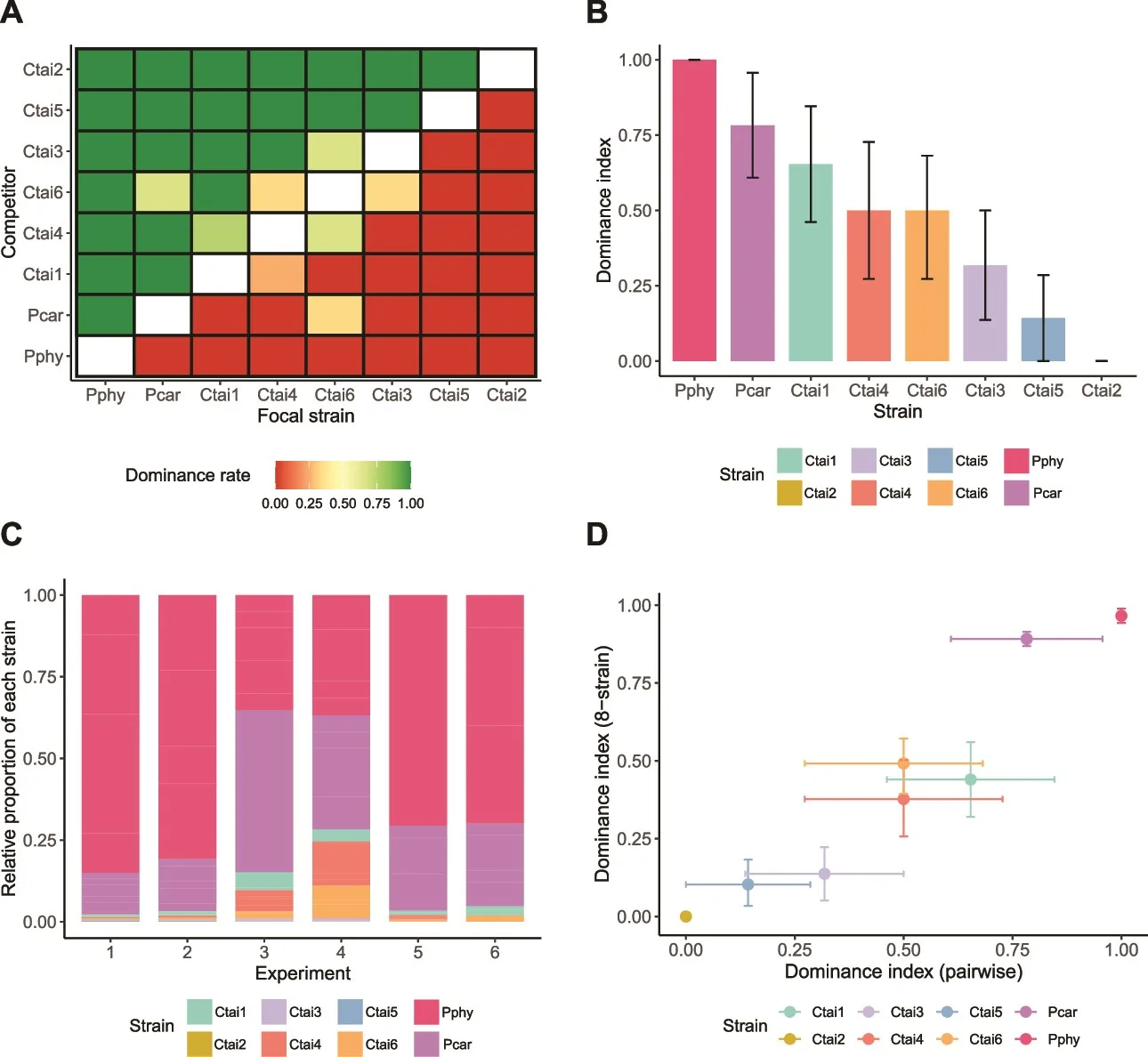

## Introduction

Rhizobia are diazotrophic soil bacteria that are able to engage in mutualistic interactions with legume plants. During these interactions, bacteria fix atmospheric nitrogen and provide ammonium to their host plant in exchange for carbon compounds derived from photosynthesis [1]. One hallmark of this symbiosis is the formation of dedicated root organs, called nodules, which house millions of intracellular symbiotic bacteria. These organs are formed after mutual recognition between the two partners, involving the exchange of chemical signals and subsequent signalling cascades [2]. In most cases, only one bacterium is trapped within the curl of a root hair, which invaginates to form a tubular structure, called an infection thread, where bacteria divide and progress towards inner root tissues [3]. At the same time, cortical root cells at the base of infected root hair actively divide to form the nodule. Bacteria are then released within nodule cells, where they differentiate into nitrogen-fixing bacteroids. During this symbiosis, mutualism (nitrogen fixation) occurs in the late stages of the interaction and is thus decoupled from the early infection process [4–7]. This creates an opportunity for nonmutualistic bacteria (e.g. bacteria that fix little or no nitrogen) to invade legume roots and benefit from the interaction without providing any benefit to their host plant [8]. In natural conditions, legumes are exposed to diverse and complex microbial communities. Multiple rhizobial strains, and other soil microorganisms, can compete or interact during the infection process, potentially influencing symbiotic efficiency [9, 10]. Such community-level interactions add an additional layer of complexity, making it challenging to characterize the relative contribution of different microbial traits on fitness and to predict symbiotic outcomes.

In rhizobia, symbiotic fitness, which we define here as the number of reproductively viable bacteria detected within plants, depends on three main phenotypic traits: (i) the proliferation and survival of bacteria in the environment surrounding the roots, (ii) the ability to induce the formation of new nodules and compete with other rhizobial strains, and (iii) the ability to infect nodule cells, survive within them and fix nitrogen. Rhizobial fitness in a given environment depends not only on the genetic characteristics of the focal strain but also on the genetic and physiological characteristics of its host plant. Indeed, legume plants employ several mechanisms to monitor and control bacterial infection throughout the symbiotic interaction [11–13]. These mechanisms can be classified depending on whether they act during pre- or postinfection processes. Pre-infection control mechanisms mainly refer to the selection of compatible rhizospheric rhizobia by the plant, leading to the initiation of nodulation. These mechanisms rely on mutual recognition processes between partners and involve, on the bacterial side, the production of Nod factors, surface polysaccharides, or secreted effectors [14]. The ability of a focal strain to form nodules on its host plant when competing with other strains present in the rhizosphere (i.e. nodulation competitiveness) involves a wider range of bacterial traits, including motility, stress resistance, and metabolic properties. This phenomenon is well known for explaining the dominance of certain strains in legume nodules [15, 16]. At the postinfection level, bacterial proliferation and survival can be modulated by sanctions and/or rewards, in the form of restricted or preferential nutrient allocation imposed by plants on bacteria, depending on the amount of fixed nitrogen provided by the different strains [11, 16–18]. Recently, a mechanism of ‘conditional sanctions’ was described in pea, during which plants allocate more resources to an intermediate fixer when it is co-inoculated with a nonfixer, than when the same strain is co-inoculated with a good fixer [19]. In addition, plant immunity can act within nodules to influence bacterial proliferation and survival [20].

Beyond direct plant–rhizobia interactions, bacterial fitness is also influenced by interactions with other members of the microbiome [9, 10]. These interactions can be direct (microbes competing or interfering with each other in the rhizosphere or within nodules) or indirect (e.g. mediated by the host plant during the nodulation or postinfection steps) and may occur at any of the different steps of the rhizobial life cycle. Examples of indirect interactions include the abovementioned conditional sanctions, or plant control of nodulation acting on strains depending on their genotypes. While the existence of direct and indirect pre-infection competitive effects affecting nodulation have long been recognized [9, 21], the analysis of postinfection interbacterial interactions has been more sporadic [19], probably because of difficulties to quantify bacterial abundance within individual nodules [9]. Yet, culture-independent methods based on quantitative Polymerase Chain Reaction (qPCR) or deep DNA sequencing [22, 23] offer tools for precise quantification of the relative influence of the different components of rhizobial fitness during symbiosis and with different levels of microbial community complexities.

Here, to understand how rhizobial fitness is affected by the presence of other legume symbionts, we quantified the effect of interbacterial interactions on rhizobial abundance during the pre- and postinfection stages of symbiosis. We used the tropical legume plant *Mimosa pudica* and a collection of nine of its rhizobial symbionts (spanning two genera and four species of beta-rhizobia). We first compared rhizobial abundances in single and pairwise inoculation experiments to identify cases where interbacterial interactions affect fitness. We then investigated at which stage of the life cycle these interactions take place. Finally, we tested whether interactions are conserved in more complex communities of six and eight strains, constituting intra- and interspecific strain assemblages, respectively. Our results showed that nodulation competitiveness is the major type of interbacterial interactions driving rhizobial fitness, with postinfection interactions making only minor contributions. Moreover, we observed that the competitive effects in pairwise inoculations were transitive, enabling the prediction of dominance ranks in more complex communities.

## Materials and methods

### Plant growth

*Mimosa pudica* seeds were produced at Laboratoire des interactions plantes-microbes-environnement in 2020, following vegetative propagation of a single maternal plant, originally coming from a commercial seed lot of Australian origin sourced from B&T World Seed, Paguignan, France. Seeds were sterilized as described in [24]. Germinated seedlings were transferred to glass culture tubes under nitrogen-free conditions. Each tube contained a slant of 20 ml of solid Fahraeus medium [25] and 40 ml of 1:4-diluted Jensen liquid medium [26]. Plants were cultivated in a growth chamber at 28°C with a 16-h light/8-h dark photoperiod. Tubes were randomly positioned within each experimental replicate to control for positional bias.

### Symbiotic phenotypes assays

Symbiotic performance of rhizobial strains was evaluated using single, pairwise, and community inoculation assays on *M. pudica* plantlets. Bacterial strains used in this study are listed in Table 1. Nine strains were used for single and pairwise assays (36 combinations of strains). Community assays consisted of either six strains (*Cupriavidus taiwanensis* strains only) or eight strains (six *C. taiwanensis* strains plus *Paraburkholderia* strains Pphy and Pcar). The strain Csp was excluded from community assays due to its near-complete absence in pairwise competitions.

**Table 1:** List of strains used in this study.

Strain ID	Description	Origin	Host plant	Genome size (bp)	Genome assembly ID	Reference
**Ctai1**	*Cupriavidus taiwanensis* LMG19424, Sm^R^	Taiwan	*Mimosa pudica*	6 476 523	GCA_000069785.1	[66]
**Ctai2**	*Cupriavidus taiwanensis* STM6021 (M1-3C2)	French Guiana	*Mimosa pudica*	6 811 355	GCA_900249815.1	[48]
**Ctai3**	*Cupriavidus taiwanensis* STM8959 (ip2.30)	Philippines	*Mimosa diplotricha*	6 705 804	GCA_900250045.1	[67]
**Ctai4**	*Cupriavidus taiwanensis* STM8969 (MAPUD 10.1)	Costa Rica	*Mimosa pudica*	6 760 360	GCA_900250015.1	[68]
**Ctai5**	*Cupriavidus taiwanensis* STM8970 (cmp52)	Texas	*Mimosa asperata*	7 026 539	GCA_900249975.1	[69]
**Ctai6**	*Cupriavidus taiwanensis* STM6160	New Caledonia	*Mimosa pudica*	7 023 887	GCA_900250125.1	[50]
**Csp**	*Cupriavidus* sp. STM8968 (AMP6)	Texas	*Mimosa asperata*	7 579 563	GCA_000426345.1	[69]
**Pphy**	*Paraburkholderia phymatum* STM815	French Guiana	*Machaerium lunatum*	8 598 258	GCA_000020045.1	[70]
**Pcar**	*Paraburkholderia caribensis* TJ182	Taiwan	*Mimosa diplotricha*	9 205 673	GCA_003028645.1	[71]
**Ctai1 ∆*nifH***	*nifH* deletion mutant from Ctai1					[6]

All bacterial strains were cultivated at 28°C on tryptone–yeast extract (TY) agar supplemented with 6 mM CaCl₂ and resuspended in sterile distilled water to an OD_600 nm_ of 0.02 (10^6^ CFU/ml). For all assays, each plantlet was inoculated 3 or 4 days postgermination with 100 μl of bacterial suspension (e.g. 10^5^ CFU per plant). For pairwise and community assays, strains were mixed in equal proportions prior to inoculation. To verify initial inoculum proportions, serial dilutions of each bacterial suspension were plated on TY agar using an EasySpiral automatic plater (Interscience), and colonies were counted after 48 h at 28°C (Supplementary Fig. 1, 2, 3A, and 4A).

Nodules were harvested 28 days postinoculation. Nodules were surface-sterilized (2.4% sodium hypochlorite, 15 min), rinsed three times with sterile distilled water, and homogenized in 1 ml of 5 mM MgSO₄ using a sterile pestle. The nodule homogenates were vortexed and centrifuged at 100 × *g* for 5 min to remove root debris. From the supernatant, 100 μl were collected, boiled at 100°C for 15 min to lyse cells, cooled on ice for 5 min, and stored at −20°C. Bacterial strain abundance within the nodule homogenate was quantified by qPCR using strain-specific primers (Supplementary Table 1). For pairwise, eight-strain, and six-strain community inoculations, bacterial abundances within nodules were normalized by the ratio of initial inoculum (shown in Supplementary Figs 1, 3A, and 4A, respectively), allowing us to control at the same time for slight deviations from a 1:1 ratio due to pipetting errors and for the expectation that plants inoculated with two, six, or eight strains should be able to support only half, one-sixth, or one-eighth of the population of each strain compared to single-strain inoculations, respectively. For shoot biomass measurements, shoot plant parts were dried at 65°C for 48 h before weighting.

### Nodulation competitiveness assays

Ten pairs of strains showing coexistence were selected to assess nodulation competitiveness. Individual nodules were harvested from a pool of 10 plants and individually crushed in flat-bottom 96-well plates. Controls were included in the remaining three wells: two wells contained one nodule from a single-inoculated plant with each strain of the tested pair and a negative control. Nodules were homogenized individually in 96-well plates in a total volume of 30 μl of 5 mM MgSO₄. From each homogenate, 10 μl was mixed with 90 μl of ultrapure water (Millipore), boiled at 95°C for 15 min, cooled on ice for 5 min, and stored at −20°C before qPCR analysis.

### Individual nodule infectivity

The bacterial load within each nodule was assessed from individual nodules using qPCR quantification. In the case of Ctai1–Ctai6 and Ctai6–Ctai4 co-inoculations, viable bacteria per nodule were assessed by plating serial dilutions of nodule crushes on TY agar plates containing 6 mM CaCl2. These samples were also analysed by qPCR to determine the identity of the strain occupying each nodule.

### Rhizosphere survival and colonization assays

We inoculated plantlets with either single strains or 1:1 mixtures. Seven days postinoculation, roots were harvested along with 10 ml of liquid Jensen medium and transferred into sterile 15 ml tubes. Samples were vortexed for 30 s to detach rhizosphere bacteria, after which roots were removed and the suspensions centrifuged at 8000 rpm for 5 min. Supernatant (9 ml) was discarded, and the bacterial pellet was resuspended in the remaining 1 ml. Samples were boiled at 100°C for 15 min, cooled on ice for 5 min and stored at −20°C for subsequent qPCR analysis.

### Experimental design and replication

To account for plant-to-plant variability in our diverse experimental settings, the replication structure of each type of assay was adjusted. For single-strain inoculation assays, nodules were harvested and pooled from individual plants and treated independently. For each strain we analysed 3 individual plants per biological replicate, with four biological replicates, for a total of 12 plants per strain. For pairwise co-inoculation and community inoculation assays (six- and eight-strain), the nodules from three plants per sample were pooled to facilitate the detection of less competitive strains, yielding one data point per biological replicate across three or four biological replicates. For single-nodule assays, the nodules from ~10 plants (~10 nodules per plant) were harvested for each of the three biological replicates, surface-sterilized together. Then, 93 individual nodules were distributed across a 96-well plate for qPCR analysis. Where comparisons were made across assay types, differences in the unit of replication were taken into account during statistical analyses. For rhizosphere assays, the rhizosphere and media of three individual plants were analysed independently, with three biological replicates per sample, for a total of nine data points per sample.

### Design of primers, standard preparation, and qPCR

Genomic DNA from each strain was extracted from liquid TY cultures using the Wizard® Genomic DNA Purification Kit (Promega). DNA concentration and purity were determined using both a NanoDrop™ spectrophotometer and a Qubit™ fluorometer. For each of the nine strains analysed in this study, a pair of strain-specific oligonucleotides targeting the *gyrB* locus was designed according to standard quality criteria, including melting temperature (Tm), Guanosine and Cytosine (GC) content, amplicon size, and sequence specificity (Supplementary Table 1).

To verify primer specificity and amplification efficiency, cross-amplification tests were performed for all primer pairs against all genomic DNA targets (50 ng/μl), and standard curves were generated from purified genomic DNA (Supplementary Fig. 5A). Standard curves consisted of four 10-fold serial dilutions ranging from 2 to 0.0002 ng/μl. Amplification specificity was confirmed by melt curve analysis at the end of each qPCR run. The slope, *y*-intercept, and coefficient of determination (*R*^2^) values of the standard curves are reported in Supplementary Fig. 5B. Each 7 μl qPCR reaction contained 3.5 μl of Takyon™ No Rox SYBR® MasterMix (Eurogentec), primers at a final concentration of 10 μM each, and 2 μl of template DNA. Amplifications were carried out on a LightCycler® 480 system (Roche) under the following cycling conditions: initial denaturation at 95°C for 3 min, followed by 40 cycles of 95°C for 10 s, 58/62°C for 15 s, and 72°C for 15 s. When required, a melting curve program was included (95°C for 10 s, 55°C for 15 s, followed by a gradual increase to 95°C). In each qPCR run, a standard curve was included using 2 ng/μl of genomic DNA. To calculate DNA copy numbers, an average standard curve was generated for each strain using all standard curves obtained, along with the known genome size of each strain (Table 1).

For experimental assays, qPCR was performed on various sample types, including nodule homogenates (in single, pairwise, and multiple inoculation) and rhizosphere samples. Frozen samples (prepared as previously described in the respective sections) were thawed on ice. A 25-fold dilution was then prepared, and 2 μl of this diluted lysate was used as template for qPCR. For samples corresponding to mixed inoculation experiments, independent qPCR runs were performed with each primer pair corresponding to each inoculated strain. When analysing qPCR results, we defined a threshold Ct value of 35, which corresponded on average (across all strains) to ~3.10^4^ DNA copies per plant. Because this quantity is below the expected bacterial content of a single nodule, samples with Ct ≥ 35 were considered below the detection threshold. For individual nodule assays, we observed that around 5% of nodules were co-infected (where both strains showed a Ct < 35). However, these nodules were discarded from subsequent analyses as we believe these are a consequence of high-density inocula used in our experiments.

### Statistical analyses

All statistical analyses were performed in R version 4.2.2 [27], using the packages dplyr [28], tidyverse [29], lme4 [30], dunn.test [31], and ggplot2 [32]. Full details on statistical analyses are provided in section 1 of the Supplementary Online Material (SOM).

## Results

### Symbiotic fitness of nine rhizobial symbionts of *M. pudica* in single inoculations

To investigate the effect of bacterial interactions on rhizobial fitness, we used a collection of nine strains that were isolated from different host plants and countries (Table 1). This collection includes six *C. taiwanensis* strains (abbreviated Ctai1–Ctai6 in the rest of the manuscript) that belong to different putative sub-species of the *C. taiwanensis* species complex [33]. In addition, we used *Cupriavidus* sp. AMP6 (Csp), and 2 *Paraburkholderia* strains: *P. phymatum* STM815 (Pphy) and *P. caribensis* Tj182 (Pcar). All strains have a similar growth rate in rich medium, with a slight reduction observed for the two *Paraburkholderia* strains (Supplementary Fig. 6).

**Figure 1:**
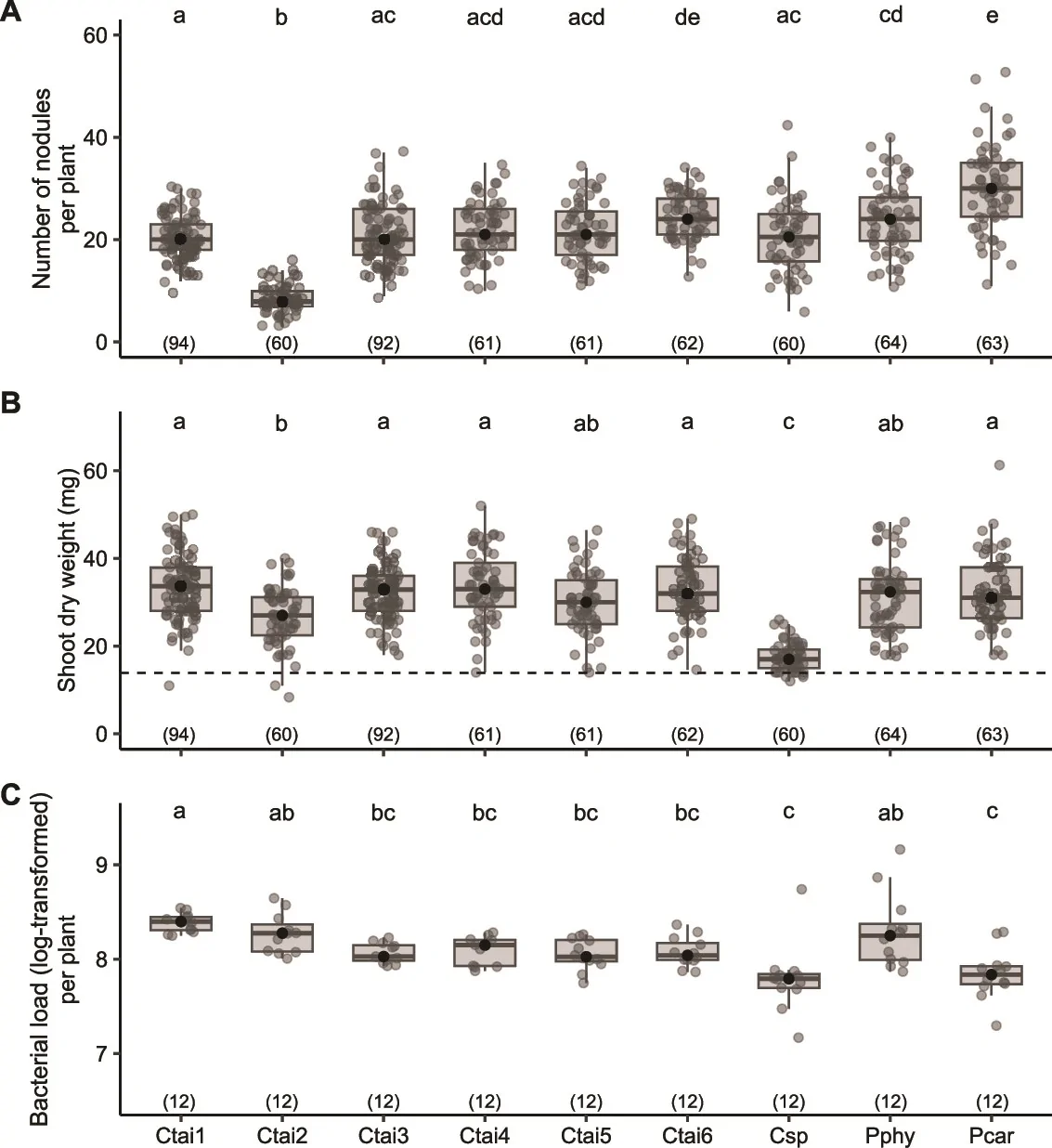
Symbiotic phenotypes of *M. pudica* symbionts in single inoculation. (A) Number of nodules per plant. (B) Plant dry weight. (C) Bacterial load per plant. Measurements were performed on plants inoculated with the different bacterial strains at 28 dpi across four independent experiments. The distributions of data points are shown in grey; black points show the medians. Sample sizes are indicated between brackets. In (B), the dotted line indicates average dry weight for noninoculated plants. Statistical analyses were performed with nonparametric Kruskall–Wallis with Dunn’s *post hoc* test. Letter groups indicate statistical significance (*P* < .05). Detailed statistical results are provided in section 2 of the Supplementary Online Material (SOM).

To establish reference measurements for each rhizobial strain without any competitor, we first assayed the symbiotic phenotypes of each strain in single-inoculation experiments. We inoculated the nine strains individually on *M. pudica* seedlings and measured the dry weight (DW) of aerial parts, as a proxy for plant fitness, and the number of nodules formed on the entire root system at 28 days postinoculation. The tested strains formed, on average, between 8.5 (for Ctai2) and 30 (for Pcar) nodules per plant at 28 dpi (Fig. 1A, SOM section 2). Almost all strains provided significant and comparable growth benefits to *M. pudica*, with average dry weights ranging from 30 (Ctai5) to 33.6 mg (Ctai4) per plant (Fig. 1B). The only exception is the strain *Cupriavidus* sp. AMP6 (Csp) for which plants’ DW was only slightly higher than noninoculated plants (17.6 vs. 13.9 mg per plant for noninoculated plants). As a proxy for bacterial symbiotic fitness, the total bacterial DNA content was measured by qPCR, using strain-specific primers (Supplementary Fig. 5; Supplementary Table 1), in pools of nodules harvested from individual plants. While this technique cannot account for changes in bacterial viability that are independent of DNA content, it enables us to monitor bacterial load in nodules from single and multiple strain inoculation experiments, eliminating the need to rely on genetic markers that could affect rhizobial fitness. The median bacterial load values were continuously distributed between ~7 × 10^7^ (Pcar and Csp) and 2.5 × 10^8^ (Ctai1) copies of bacterial genomes per plant (Fig. 1C). Overall, these results show that all strains effectively nodulate *M. pudica* and all, with the exception of Csp, are efficient mutualistic symbionts of the genotype used in our experiments.

### Pairwise inoculation experiments reveal a high prevalence of interbacterial negative interactions

**Figure 2:**
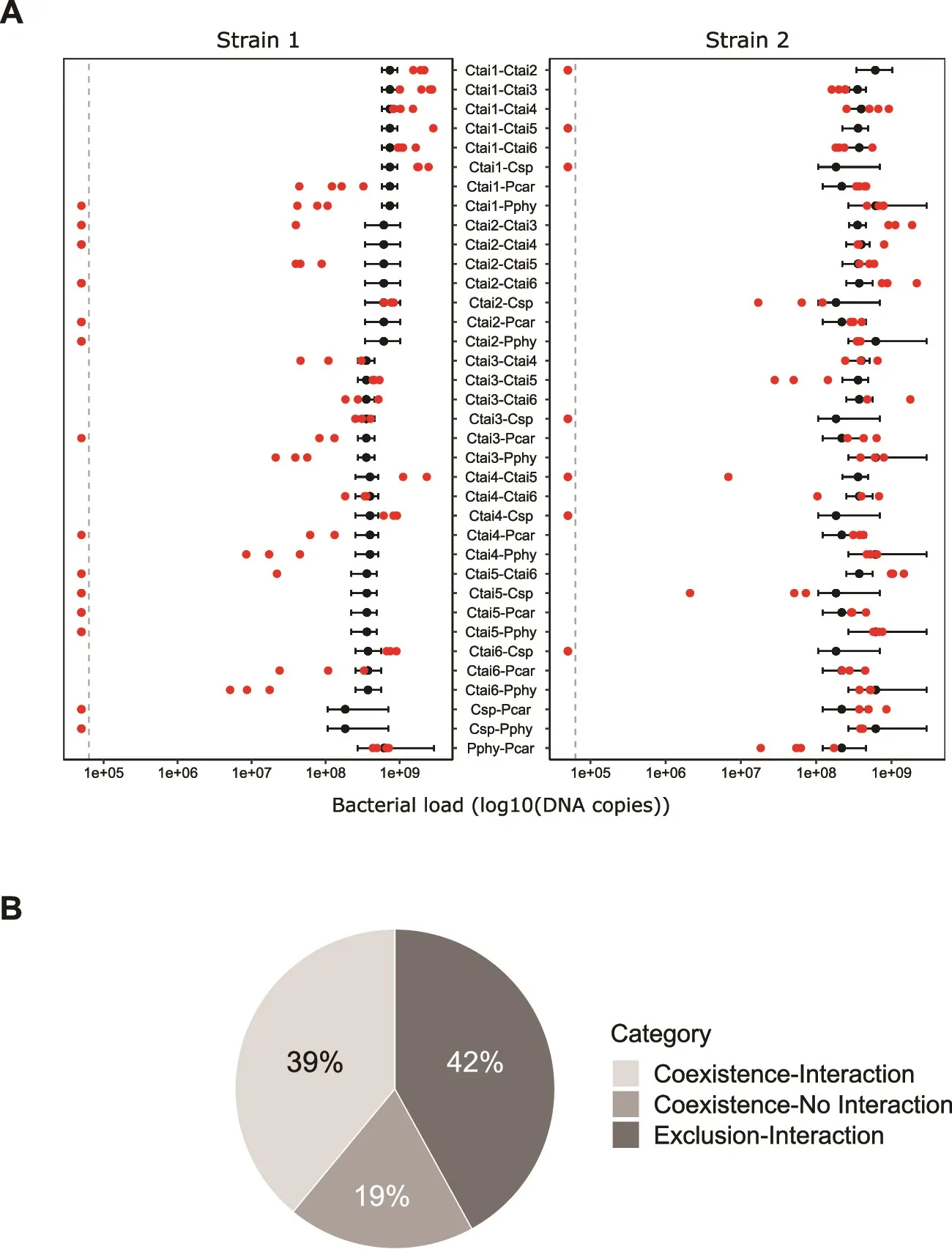
Identification of interactions during symbiosis for 36 pairs of rhizobial strains. (A) Comparison of bacterial loads in nodules from single and pairwise inoculations. Red dots show pairwise inoculation data (*n* = 3 or 4). Black dots and error bars represent the mean and 95% confidence intervals, respectively, of bacterial loads predicted from single inoculations (from bootstrap iterations). The dotted line indicates the limit of detection. (B) Summary of the classification of pairwise inoculations according to the existence and type of interbacterial interaction.

To detect possible interbacterial interactions occurring during symbiosis within our strain collection, we performed 36 pairwise co-inoculations, representing all possible combinations of the nine strains, and measured the same symbiotic properties as before (number of nodules, plant dry weight, and bacterial abundance within nodules). Overall, no systematic bias on plant growth could be detected in pairwise compared to single-inoculation experiments (Supplementary Fig. 7), showing that bacterial competition has negligible effects on plant development. However, comparing bacterial abundances in these two settings revealed numerous cases where bacterial loads were different from those expected from single-inoculation data (Fig. 2A and SOM section 1). We considered the bacterial load to be significantly different in pairwise compared to single-inoculation experiments when all the replicate data points from the pairwise comparisons fell outside of the 95% confidence interval predicted from the single-inoculation data. In other words, bacterial loads were not deemed as different when at least one pairwise data point fell within the 95% confidence interval. This strict definition was adopted to increase the robustness (while decreasing sensitivity) of our screen aiming at identifying large-effect interbacterial interactions affecting fitness. For each pair of strains, we then classified the outcome of inoculation in three categories: (i) co-existence of the two strains without interaction, when both strains’ load was not different from single inoculation, (ii) co-existence with interaction, when the abundance of at least one strain was (positively or negatively) affected by the presence of the other strain, and (iii) interaction with exclusion, when the abundance of one strain fell below the detection limit in all replicates. Using this classification, evidence for interstrain interactions was found in 81% of the cases (29 out of 36 pairs of strains; Fig. 2B). Out of these 29 cases, extinction of one strain was observed in 15 pairs. These results show that interactions between rhizobial strains are frequent and can have major effects, by leading to the extinction of one strain. In the 14 other cases of interactions, both strains remained in co-existence.

**Figure 3:**
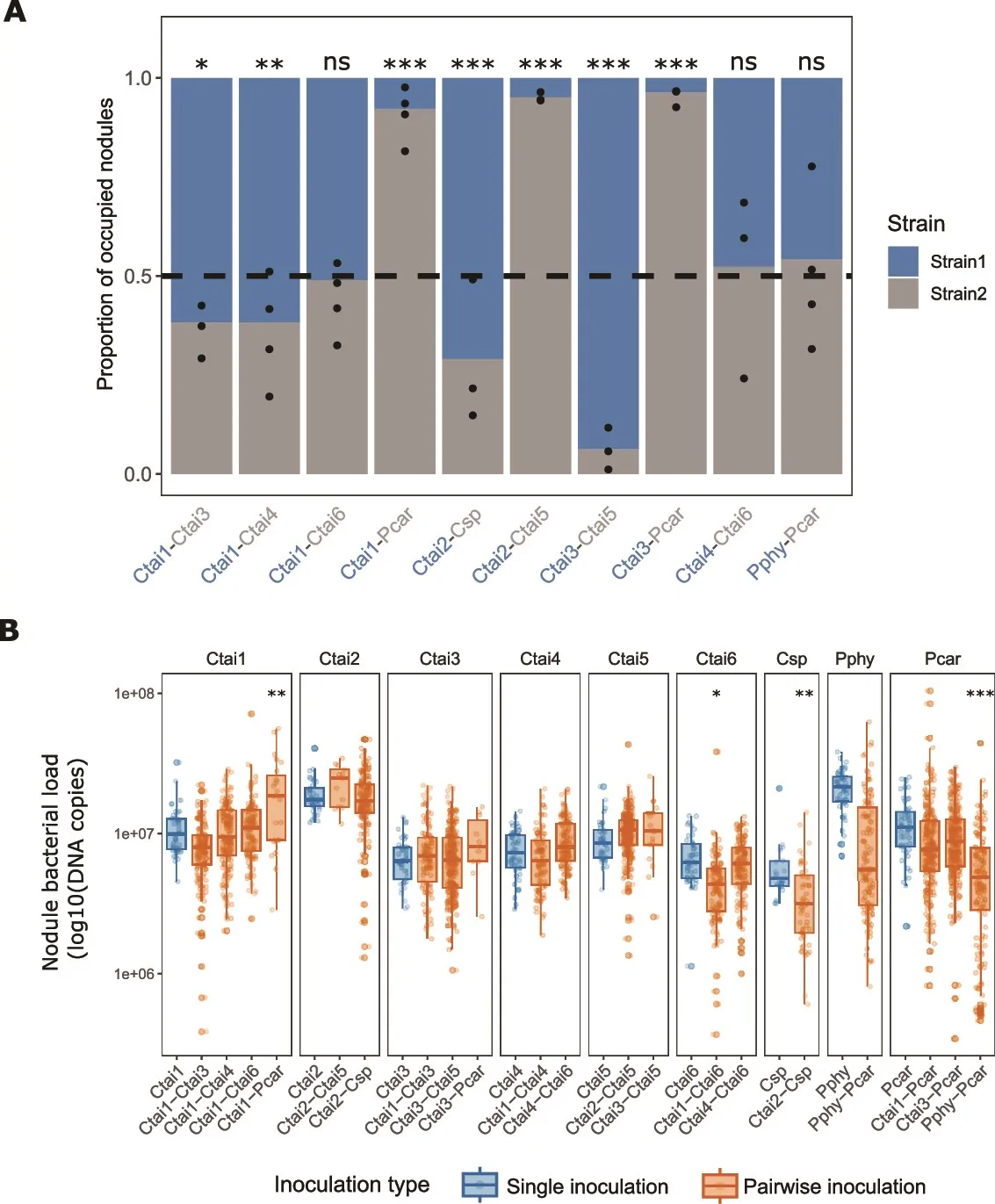
Characterization of interactions across different steps of the symbiotic life cycle for 10 pairs of strains. (A) Proportion of nodules occupied by each competing strain in pairwise inoculations. Initial proportions of each strain in the inoculum are shown in Supplementary Fig. 2. Asterisks indicate significant deviations from the expected 50% (equal occupancy) in co-inoculation experiments, reflecting differences in nodulation competitiveness between strains. Statistical significance was assessed using a GLMM with a binomial distribution and logit link function. Detailed statistical results are provided in section 3 of the SOM. (B) Comparison of bacterial load within individual nodules from single and co-inoculated plants. Each panel shows data for the quantification of one focal strain (indicated above each panel) and represents data from plants inoculated only with this focal strain (blue boxplots) or co-inoculated with another strain (orange boxplots, with the identity of co-inoculated strains indicated below the plots). Statistical significance was assessed using a GLMM with a Gamma distribution and logarithmic link function. Detailed statistical results are provided in section 4 of the SOM. On both plots, ^*, **^, and ^***^ indicate a *P*-value below .05, .01, or .001, respectively.

### Interstrain interactions predominantly occur at the nodulation step

We then sought to identify the step of the symbiotic life cycle at which these interactions occur. In our simplified experimental system, symbiotic fitness of a focal rhizobial strain is dependent on both the number of nodules formed by this strain on a plant and the number of bacteria per nodule. In the pairwise inoculation tests described above, the 15 cases of interactions where one strain was completely excluded were likely due to the strong nodulation competitiveness of the remaining strain, as we calculated that even one nodule colonized by one strain could have been detected by qPCR (SOM section 1).

To assess the robustness of the first screen of interactions and gather a more complete view on the role of nodulation competitiveness in our experiments, we selected 10 pairs of strains showing co-existence in pairwise inoculations (Fig. 3A). Among these 10 pairs, 6 had been classified as cases of interactions, while the 4 others (Ctai1–Ctai4, Ctai2–Csp, Ctai4–Ctai6, and Pphy–Pcar) showed similar abundances in single and pairwise inoculations (when correcting for total plant carrying capacity, see SOM section 1). Among the six pairs of interacting strains, statistically significant differences in nodulation competitiveness were observed five times (Ctai1–Ctai3, Ctai1–Pcar, Ctai2–Ctai5, Ctai3–Ctai5, Ctai3–Pcar). In all these cases, the strain with the highest nodulation competitiveness also had highest relative fitness (Figs 2A and 3A), suggesting that nodulation competitiveness contributes to the observed unequal fitness. In the sixth case (Ctai1–Ctai6 pair), both strains showed similar nodulation competitiveness, suggesting that interactions occur at the postinfection level. When considering pairs that had been classified as noninteracting, we confirmed that no significant differences in nodulation competitiveness occurred for Ctai4–Ctai6 and Pphy–Pcar. On the contrary, Ctai1 and Ctai2 had slightly higher nodulation competitiveness than Ctai4 and Csp, respectively. We note, however, that although the Ctai2–Csp pair had been classified as noninteracting, a decreased in Csp abundance was observed (Fig. 2A). Overall, differences in nodulation competitiveness likely explain at least 69% of rhizobial interactions detected in pairwise inoculations (20 cases out of 29).

Variations in nodulation competitiveness can be caused either by differential growth or survival in the rhizosphere, and/or by preferential compatibility between symbiotic partners at the early stages of the interaction. To investigate which of these two mechanisms might be responsible for the effects observed above, rhizospheric bacterial populations were collected from the root surface and surrounding liquid medium of single and pairwise inoculated plants at 7 days postinoculation, when the first nodules became visible. In single inoculations, median bacterial loads in the rhizosphere varied between 10^5^ and 5 × 10^6^ DNA copies per millilitre of medium (Supplementary Fig. 8A). Except for Pcar, strains with high nodulation competitiveness (e.g. Pphy, Ctai1) did not have a markedly improved survival in the rhizosphere and, vice versa, some poor competitors (e.g. Ctai2) had a relatively high survival in the rhizosphere. However, in pairwise inoculations, a significant positive correlation was found between the proportion of bacterial loads in the rhizosphere and the proportion of occupied nodules (Supplementary Fig. 8B; Spearman correlation test, rho = 0.67, *P* = 4.4 × 10^−6^). The overall correlation between these two traits suggests that high competitiveness in the rhizosphere might underpin high nodulation competitiveness for a number of bacterial pairs. However, significant outliers were also detected, with strains showing high nodulation competitiveness despite being outcompeted in the rhizosphere. In these cases, the effect of survival in the rhizosphere is likely insignificant, and the difference in nodulation competitiveness between the two strains must instead be linked to direct plant–bacteria signalling compatibility.

### Evidence for postinfection control mechanisms

So far, we have analysed pre-infection competition effects occurring before the establishment of an effective mutualistic symbiosis. Once mature nodules are formed, postinfection interactions may also contribute to modify rhizobial fitness during pairwise inoculations. The pairwise nodulation competitiveness assays described previously also allowed us to measure bacterial abundances in individual nodules and compare these values to those found in single inoculations (Fig. 3B). In most cases, bacterial abundances in individual nodules were similar in nodules arising from single- or pairwise-inoculated cases. However, significant differences were observed in four cases. The abundance of Ctai1 increased when co-inoculated with Pcar. In the remaining three cases, co-inoculation led to a decrease in the abundance of one strain (Ctai6, Csp, and Pcar, when co-inoculated with Ctai1, Ctai2, and Pphy, respectively).

**Figure 4:**
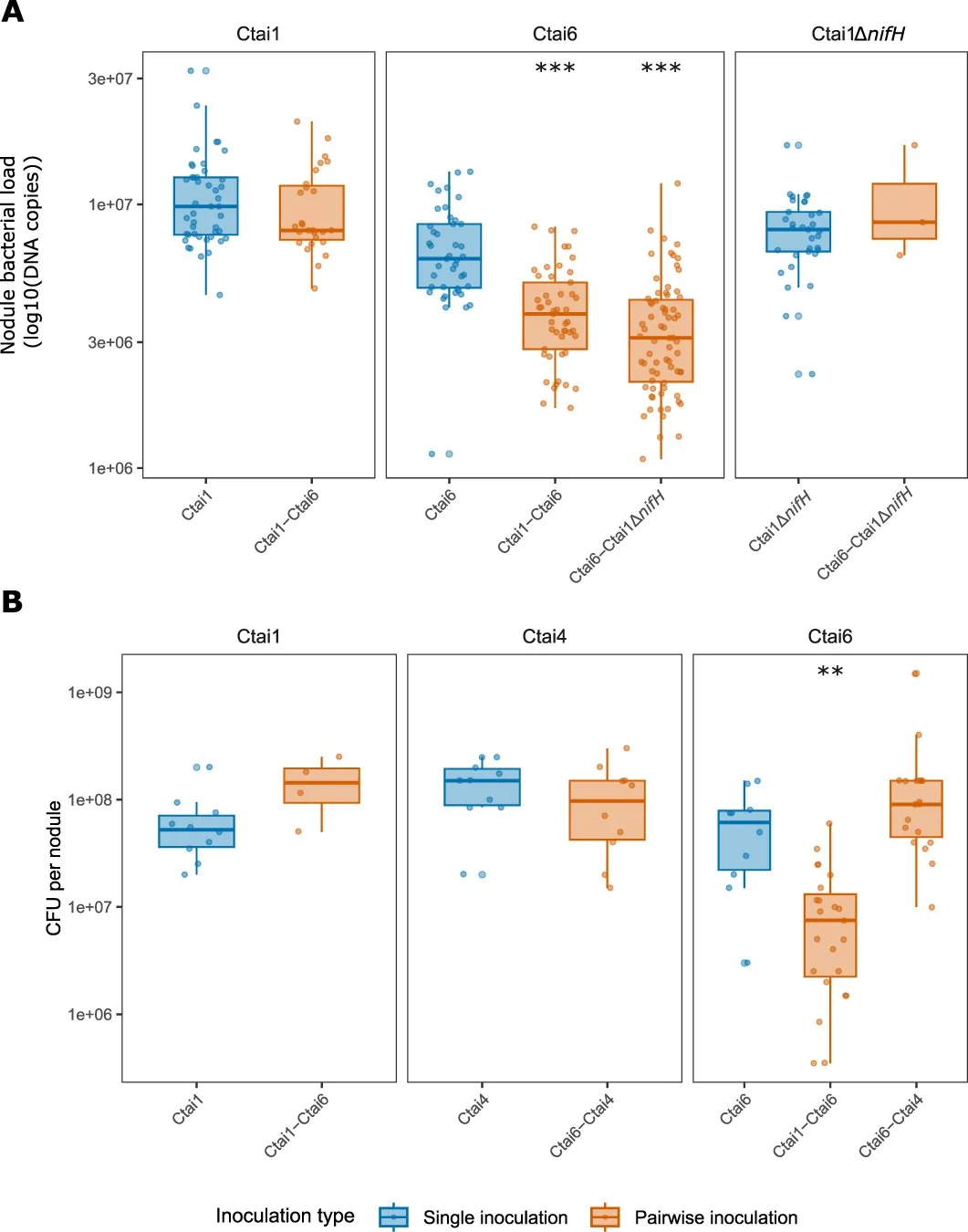
A specific postinfection interaction between Ctai1 and Ctai6 decreases fitness of Ctai6 independently of nitrogen fixation. (A) Bacterial fitness in individual nodules evaluated by qPCR. (B) Bacterial fitness in individual nodules evaluated by dilution plating (CFU counts). Each panel shows data for the quantification of one focal strain (indicated above each panel) and represents data from plants inoculated only with this focal strain (blue boxplots) or co-inoculated with another strain (orange boxplots, identity of co-inoculated strains indicated below the plots). Statistical significance was assessed using Student’s *t-*test. On both plots, ^**^ and ^***^ indicate a *P*-value below .01 or .001, respectively. In (A), *n* ≥ 27 except for Ctai1 ∆*nifH* in the Ctai6-Ctai ∆*nifH* mix where *n* = 3. In (B), *n* = 10 for single inoculations and *n* ≥ 4 for pairwise inoculations.

To further investigate conditional postinfection control in *M. pudica*, we focused on the Ctai1–Ctai6 pair since the fitness and nodulation measurements for these strains showed low variability, and their equal nodulation competitiveness facilitates the recovery of individual nodules from each strain when performing fitness measures in pairwise inoculations. In this pair of strains, Ctai1 caused a moderate (less than two-fold) reduction in Ctai6 load (Fig. 3B). The interaction between Ctai1 and Ctai6 seems specific, since Ctai1 did not cause a reduction in the load of Ctai3 or Ctai4, and the load of Ctai6 was not reduced when co-inoculated with Ctai4. Another set of experiments was performed to confirm and further explore this phenomenon. To test if conditional sanctions could explain this interaction, Ctai6 was co-inoculated with a non-fixing Δ*nifH* mutant of Ctai1 [6]. A similar decrease in Ctai6 abundance was observed when it was co-inoculated with the *nifH* mutant or with the WT Ctai1 strains (Fig. 4A), showing that this post-infection interaction effect was not linked to the nitrogen fixation level of the competing strain. Moreover, to confirm that the reduction in Ctai6 bacterial load, as measured by qPCR, indeed corresponds to a reduction in the number of viable bacteria, we measured the number of colony-forming units (CFUs) in individual nodules from single or co-inoculated plants (Fig. 4B). We observed that the median number of viable Ctai6 bacteria in nodules was ~10 times lower during pairwise inoculation with Ctai1 than when inoculated alone or co-inoculated with Ctai4, showing that qPCR data, probably by detecting DNA of nonviable bacteria, underestimate the viability loss of rhizobia within nodules. These results confirm the specific and marked reduction in Ctai6 fitness during pairwise inoculation with Ctai1.

### Transitivity in rhizobial interactions is conserved in more complex communities

**Figure 5:**
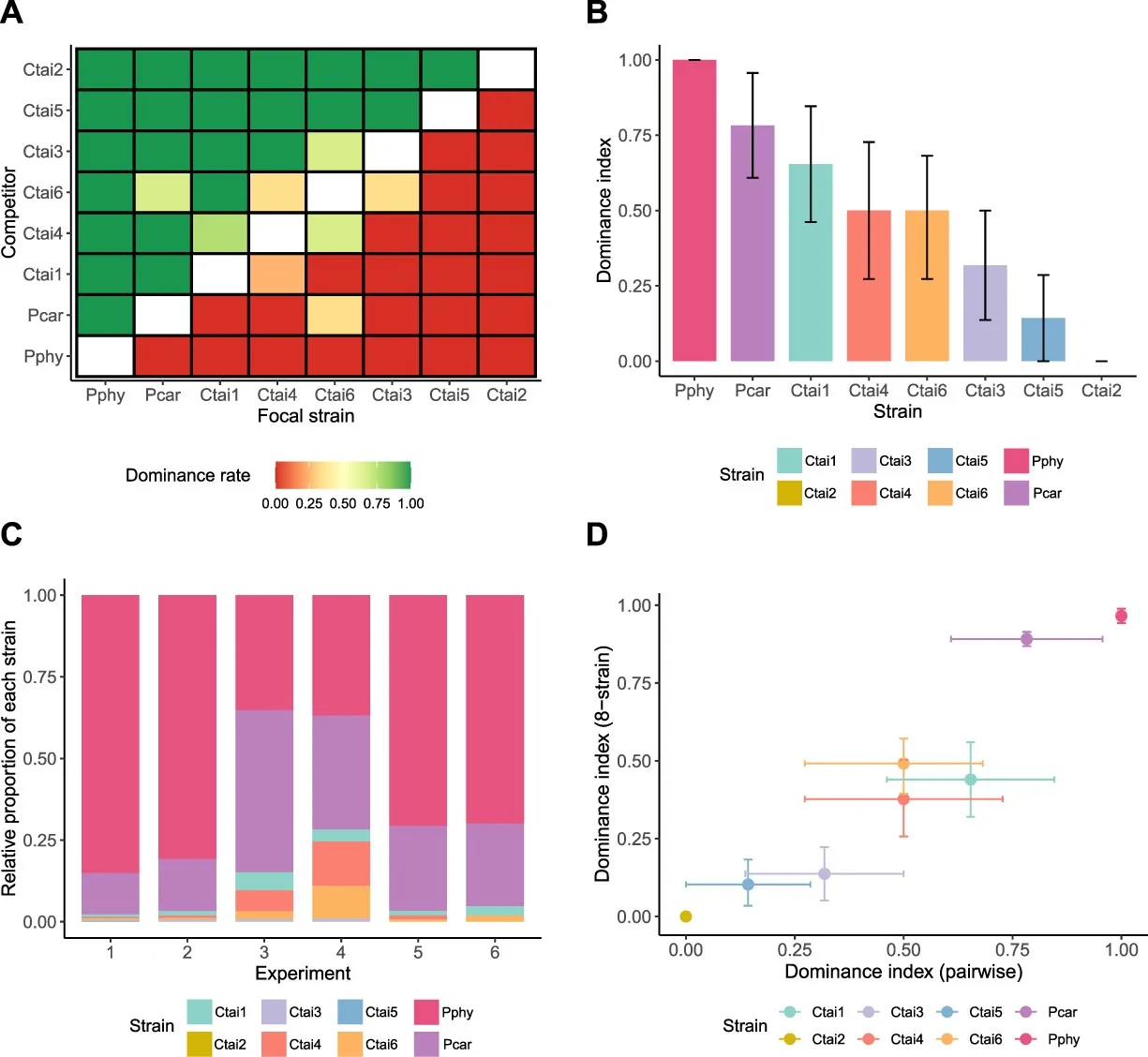
Transitivity in pairwise interactions is conserved in more complex communities. (A) Dominance rate of each focal strain in pairwise co-inoculation tests. For each pair of strains, the proportion of experiments (out of three or four independent experiments) in which the focal strain is more abundant than its competitor is shown as a colour gradient. (B) Dominance index calculated as the average of dominance rates in all pairwise inoculation data. (C) Relative frequency of strain abundances in eight-strain communities. Data from six independent experiments are shown. (D) Dominance index from eight-strain communities plotted against dominance index from pairwise inoculations.

A current challenge in microbial ecology is to assess to which extent the results of pairwise interaction studies are relevant for communities comprising numerous strains or species. Indeed, pairwise interactions often fail to predict strain abundance at a given time point in more complex microbial communities due to the existence of intransitive or higher-order interactions [34, 35]. To test for the existence of intransitive interactions, we first re-analysed bacterial fitness data from pairwise inoculation tests (shown in Fig. 2) by analysing, across all experiments, the frequency at which each strain was more abundant than its competitor (Fig. 5A). We found that the network of interactions only consists of transitive interactions, even if competition outcomes were occasionally inverted in some individual replicates of given pairs (e.g. Ctai3–Pcar, Ctai6–Pcar, Ctai1–Ctai4, Ctai3–Ctai6, and Ctai4–Ctai6). This result prompted us to test if this dominance hierarchy was conserved in communities comprising more than two members. To do so, we first computed a ‘dominance index’, ranking strains according to their average dominance in pairwise competitions (Fig. 5B, see SOM section 1 for details on index calculation). Next, we performed six independent experiments to measure relative strains’ abundances in a community comprising eight strains (Fig. 5C). We excluded the Csp strain from this community, as it went extinct in six cases out of eight in pairwise experiments (Fig. 2). We verified that no systematic bias on plant growth could be detected in community inoculations compared to single-inoculation experiments (Supplementary Fig. 9). These community assays showed that the two *Paraburkholderia* strains largely dominated this eight-strain community, since together they accounted for ≥95% of the final populations in four experiments (Experiments 1, 2, 5 and 6), and 72% or 85% of the total populations in the other two experiments. It is interesting to note that Pphy alone represented ≥70% of the community in the four experiments showing the highest proportion of *Paraburkholderia* strains. Most *Cupriavidus* strains were not completely excluded, and their total and relative abundances fluctuated among experiments. Ctai5 and Ctai2 were rarely or never detected, respectively. Within each experiment, variability between replicates (each consisting of a pool of three plants) was moderate (Supplementary Fig. 10), suggesting that between-experiment variability may originate from inoculum preparation or small uncontrolled variations in plant culture conditions. Further analysis of the origin of this variability is beyond the scope of this study but would represent a worthwhile follow-up of this work, as it was shown in other systems that small physiological differences between host or microbial individuals, or spatial structure of the environment, can lead to significant variations in the outcome of competitive infections [36–38]. To compare the results from the eight-strain communities to the pairwise inoculations, we first calculated a ‘dominance index’ in these eight-strain communities, similar to the one calculated for pairwise inoculations (SOM section 1). A positive correlation was observed between the two indexes (median rho = 0.93; 95% CI: 0.79–1.00; Spearman correlation test), showing that the hierarchy of strain dominance is conserved in the two types of experiments (Fig. 5D). High-ranking strains in the pairwise competitions (Pphy, Pcar, and to a lesser extent, Ctai1 and Ctai4) also robustly dominate in eight-strain communities. Similar results were obtained in a community comprising only the six *C. taiwanensis* strains. Indeed, Ctai1, Ctai4, Ctai6, and to a lesser extent Ctai3, were always recovered at relatively high frequency from these communities (Supplementary Figs 11A and 12), but the dominant strain varied across replicate experiments (Ctai1, Ctai6, or Ctai4). Ctai5 was barely present, while Ctai2 was always absent. Dominance indexes calculated in this community were also positively correlated with the dominance indexes calculated from pairwise inoculations (Supplementary Fig. 11B; median rho = 0.94; 95% CI: 0.77–1.00; Spearman correlation test). Overall, these results show that symbiotic fitness is relatively robust and consistent across different levels of community complexity, and that higher-order interaction effects are either absent or weak in these communities.

## Discussion

Within complex host-associated microbial communities, numerous factors influence the fitness of microbial members, including direct microbe–microbe interactions [9, 10, 39] and indirect host-mediated control mechanisms [11, 40]. Here, our aim was to investigate how the presence of other rhizobia affects directly or indirectly the fitness of a focal strain during its interaction with a legume plant.

We showed that interactions affecting fitness are extremely common when several strains co-exist. These results support the view that multi-strain inoculations are needed for a pertinent evaluation of rhizobial fitness [23, 41], as diverse rhizobial strains generally co-exist in soils [42]. Going further, analysing the different components of rhizobial fitness showed that nodulation competitiveness is the main source of interaction between rhizobia, being more frequent and generally stronger than postinfection effects. Again, this observation is consistent with previous results. Among *M. pudica* symbionts, *Paraburkholderia* strains, and *P. phymatum* STM815 (Pphy, in our study) in particular, are known to be highly competitive [43–47], even if they may be outcompeted by *Cupriavidus* strains under specific soil conditions (alkaline pH, presence of heavy metals) or on certain *M. pudica* genotypes [45, 48–51]. Interestingly, the analysis of transcriptomic responses of *P. phymatum* STM815 and *C. taiwanensis* LMG19424 to root exudates of *M. pudica* showed that STM815 had a broader response than LMG19424 and included numerous genes known to be involved in plant–bacteria interactions [52]. These results suggest a better adaptation of STM815 to the rhizosphere of *M. pudica* and are in line with the ancient evolutionary history of *P. phymatum* as a *Mimosa* symbiont [53]*.* Previous work has shown that the type-6 secretion system (T6SS) of *P. phymatum* STM815 is a major determinant of nodulation competitiveness against other *Paraburkholderia* strains, as well as an efficient antimicrobial weapon against these strains, strongly suggesting that direct antibacterial activity is responsible for high nodulation competitiveness [54]. However, in these experiments, the T6SS mutant remains a very good nodulation competitor, indicating that other properties of *P. phymatum* STM815 are involved in high competitiveness. These properties are likely important in our experiments, as we did not detect any consistent reduction in rhizospheric populations of rhizobia competing against *P. phymatum* STM815, although *P. phymatum* was extremely competitive in symbiosis against all strains tested.

A strong motivation for the detailed analysis of fitness in individual nodules of co-inoculated plants was the recent discovery of conditional sanctions in peas, which modifies bacterial loads within nodules depending on the relative levels of nitrogen provided to the plant by the two competing strains [19]. Our experiments on *M. pudica* detected a few cases of such indirect interactions, but they were rare and their effect was generally minor compared to nodulation competitiveness. In one case (Ctai2–Csp), our results are consistent with a mechanism of conditional sanctions, since the fitness of the weakest mutualist, Csp, is decreased when co-inoculated with Ctai2. In another case (Ctai1–Ctai6), we ruled out the involvement of nitrogen fixation in the establishment of conditional postinfection control. Much work remains to be done to fully characterize this phenomenon, investigate its ecological relevance in natural settings, and identify the underlying molecular mechanisms. An interesting observation, however, is that the decrease in Ctai6’s fitness seems more drastic when considering CFUs recovered from nodules than qPCR data (Fig. 4). This suggests that not only the total number of Ctai6 cells but also their viability is severely altered during competition with Ctai1. Although *C. taiwanensis* bacteroid populations are not fully terminally differentiated in *M. pudica* nodules [55], it is likely that some processes specifically controlling Ctai6 bacterial viability and survival (e.g. involving antimicrobial peptides or other defence responses, and that do not completely degrade DNA) are activated in these nodules [20]. It also reveals a limit of DNA-based quantifications of bacterial fitness that may overlook processes involving differences in viability.

Finally, we observed a robust transitive dominance hierarchy among our strains, both in pairwise inoculations and in more complex communities comprising six or eight strains. This finding suggests that, under our experimental conditions, higher-order interactions play a limited role in determining community composition and that pairwise competitive fitness can be used to predict the outcome of more complex communities. This understanding of how microbe–microbe interactions shape complex microbial communities is a current major challenge for microbial ecology [56], with some studies detecting higher-order interaction effects [34, 57–59] while others do not [60–63]. Our results are consistent with Burghardt *et al*., who reported that, in the *Medicago truncatula–Sinorhizobium meliloti* symbiosis, increasing inoculum complexity to up to 68 strains had only minor effects on relative strain rankings, suggesting that competitive hierarchies are largely maintained across community contexts [64]. In addition, Montoya *et al*. studied pairwise interactions between eight *Mesorhizobium* strains during symbiosis on *Acmispon wrangelianus* and showed that these interactions were largely transitive, even if some instances of intransitivity were also observed [65]. Consequently, the trend emerging from studies performed on diverse legume–rhizobia symbiotic associations is that competitive hierarchies among pairs of strains can provide reliable predictions of community composition across increasing levels of complexity. A future goal for microbial ecology will be to understand why certain microbial communities are less likely to exhibit intransitive and higher-order interactions than others, and to evaluate the role of eukaryotic hosts in these processes.

## Supplementary Material

Supplementary_material_ycag210

## Data Availability

All data and code generated during this work are available on the Data INRAE platform (doi:10.57745/TPHBT3).
