# Supplementary material for: Transitive interactions among rhizobia determine their symbiotic fitness
Source: ISME Commun. 2026 Jul 21;6(1):ycag210. doi: 10.1093/ismeco/ycag210 (PMC13557124; doi:10.1093/ismeco/ycag210)
Supplement: Supplementary file 1 [file supplementary_material_ycag210.zip › SELECT_SOM_final_ycag210.docx]

**Supplementary online material (SOM) for the manuscript:**

**Transitive interactions among rhizobia determine bacterial fitness during their interaction with the legume plant *Mimosa pudica***

Authors:

Margarita Granada Agudelo, Bryan Ruiz, Jean-Baptiste Ferdy, Delphine Capela, Philippe Remigi

**This document presents the detailed methods and outputs for statistical analyses performed in the manuscript. It contains 4 sections:**

**SOM section 1: Supplementary material and methods: Statistical analyses of symbiotic traits**

**SOM section 2: Pairwise comparisons of nodule number, dry weight and bacterial abundance among strains shown in Figure 1**

**SOM section 3: Model estimates of the statistical model fitted to the nodule occupancy data presented in Figure 3A**

**SOM section 4: Model estimates of the statistical model fitted to the bacterial abundances in single nodules presented in Figure 3B**

**SOM section 1: Supplementary material and methods: Statistical analyses of symbiotic traits**

All raw and transformed data generated during this work can be accessed on the data INRAE platform at the following url: <https://doi.org/10.57745/TPHBT3>. All experiments performed during this work were attributed a unique identifier, ranging from S17 to S63 (with gaps). These identifiers are used in some supplementary figures and are indicated in the ‘Expt’ columns of the data tables available on the data INRAE platform.

*Symbiotic phenotype comparisons in single inoculations*

Kruskal–Wallis rank-sum tests were used to assess variations in symbiotic phenotypes (shoot dry weight, nodule number, and bacterial abundance in nodules) across strains in single inoculation experiments. When significant differences were detected, post hoc pairwise comparisons were conducted using Dunn’s test with Bonferroni correction to adjust for multiple testing. Data for bacterial abundances within nodules comes from four independent experiments, each containing 3 individual plants (12 plants analysed in total). Each measurement corresponds to bacterial abundance found in all pooled nodules from one single plant. For dry weights and numbers of nodules, at least 60 individual plants were analysed, across multiple experiments. Each sample corresponds to one individual plant.

*Pairwise combination screen*

To evaluate inter-strain interactions in 36 pairwise combinations, we first generated the distribution of predicted strain abundance under the null ‘no-interaction’ hypothesis by bootstrapping experimental data values of bacterial abundances per plant in single inoculation tests (n = 1000 iterations per strain, sampling with replacement). For each strain, the expected abundance in co-inoculation was calculated by summing three randomly chosen single-inoculation values (to take into account the fact that pairwise inoculation data came from 3 pooled plants while single inoculation data came from individual plants), and 95% confidence intervals (CIs) were computed.

Next, we performed competition experiments to obtain observed abundance values for each pair of co-inoculated strains. To correct for deviations from the intended 1:1 inoculum ratio and for the reduced per-strain carrying capacity under pairwise competition, observed strain abundances were normalized by dividing each strain’s abundance by its initial inoculation proportion (typically ~0.5), thereby scaling abundances relative to their starting representation. The proportions of each strain in the initial inoculum of each experiment are shown in Supplementary Figure 1. For each of the 36 pairs of strains tested, three or four independent experiments were realised. In each case, the nodules of three individual plants were pooled before qPCR quantification of the abundance of each strain with strain-specific primers. A total of 13 independent experiments was needed to gather the data for all pairs of strains.

Finally, we compared the observed abundance values from each pairwise inoculation with the predicted CIs. We classified a pair of strains as “interacting” when all observed abundance values of at least one of the two strains fell outside of the CI predicted from single inoculation data. Among the cases of interactions, outcomes were classified as “dominance” when only one strain was detected in the experiment (having driven the other competing strain to extinction), or as “coexistence” when both strains were detected.

*Nodulation competitiveness assays*

Nodulation competitiveness in pairwise co-inoculations was analysed in 3 independent experiments for each of the 10 pairs of strains tested in this assay. In each experiment and for each pairwise inoculation, 93 nodules were harvested from 10 co-inoculated plants. A total of 10 independent experiments were required to gather all these replicates. Nodulation competitiveness data were analysed using a binomial generalized linear mixed-effects model (GLMM). The response variable was the proportion of nodules occupied by a focal strain modelled as a binomial outcome (number of nodules occupied vs. not occupied). The identity of the competing strain pair was included as a fixed effect. To account for variation among independent experimental runs, experiment batch was included as a random intercept.

*Comparison of pairwise and community inoculations*

A dominance index was calculated for each strain based on its overall performance in all pairwise co-inoculations. More specifically, we recorded whether a strain won or lost against its competitor. Winners were defined as the strain reaching a higher absolute abundance than its competitor and were assigned a score of 1; losers were assigned a score of 0. To account for experimental variability, we performed a bootstrap approach by resampling, from pairwise inoculation data, the abundance values of a focal strain and of one of its competitor, and giving a score to each strain based on their relative abundance (n = 1000 iterations, sample with replacement). For each strain, the dominance index was defined as the mean score across all iterations of bootstrapped pairwise competitions. A similar bootstrap procedure (n = 1000 iterations, sampling with replacement) was used to compute dominance indexes from the 6-strain and 8-strain community inoculations. Because these experiments were performed in batches, bootstraps were generated by sampling replicates within each batch to account for the experimental structure. In these community assays, observed experimental values were obtained by the same qPCR-based quantification approach as in pairwise co-inoculations. For these community inoculations, data comes from 6 independent experiments, each comprising 4 or 5 replicate pools of nodules each originating from three individual plants. To account for potential variation in inoculum composition in these experiments, observed strain abundances were normalized by dividing the measured abundances by their initial inoculation ratio. The proportion of each strain in the initial inoculum of each experiment can be seen in Supplementary Figures 3 and 4.

To assess whether dominance indexes measured in pairwise inoculations could explain the strains performance in more complex communities, we computed a Spearman rank correlation between the pairwise and community dominance indexes for each bootstrap iteration. This procedure generated a distribution of correlation coefficients, which we used to evaluate whether strain rank observed in pairwise inoculations were maintained in 6-strain or 8-strain communities.

**SOM section 2: Pairwise comparisons of nodule number, dry weight and bacterial abundance among strains shown in Figure 1**

**Pairwise comparisons were performed using Kruskal–Wallis tests followed by Dunn’s post-hoc tests with Bonferroni correction .**

***Number of nodules:***

**The Kruskal–Wallis test revealed a significant effect of the strain on nodule number (χ^2^ = 227.73, df = 8, P < 2.2 × 10^-16^).**

| Comparison | Z statistic | Adjusted P value |
| --- | --- | --- |
| Csp – Ctai1 | 0.36 | 1.000 |
| **Csp – Ctai2** | **7.72** | **<0.001** |
| Csp – Ctai3 | −0.20 | 1.000 |
| Csp – Ctai4 | −0.90 | 1.000 |
| Csp – Ctai5 | −0.87 | 1.000 |
| **Csp – Ctai6** | **−3.41** | **0.011** |
| **Csp – Pcar** | **−6.07** | **<0.001** |
| Csp – Pphy | −2.57 | 0.185 |
| **Ctai1 – Ctai2** | **8.17** | **<0.001** |
| Ctai1 – Ctai3 | −0.62 | 1.000 |
| Ctai1 – Ctai4 | −1.36 | 1.000 |
| Ctai1 – Ctai5 | −1.32 | 1.000 |
| **Ctai1 – Ctai6** | **−4.14** | **<0.001** |
| **Ctai1 – Pcar** | **−7.08** | **<0.001** |
| Ctai1 – Pphy | −3.21 | 0.024 |
| **Ctai2 – Ctai3** | **−8.69** | **<0.001** |
| **Ctai2 – Ctai4** | **−8.65** | **<0.001** |
| **Ctai2 – Ctai5** | **−8.56** | **<0.001** |
| **Ctai2 – Ctai6** | **−11.20** | **<0.001** |
| **Ctai2 – Pcar** | **−13.88** | **<0.001** |
| **Ctai2 – Pphy** | **−10.41** | **<0.001** |
| Ctai3 – Ctai4 | −0.79 | 1.000 |
| Ctai3 – Ctai5 | −0.76 | 1.000 |
| **Ctai3 – Ctai6** | **−3.56** | **0.007** |
| **Ctai3 – Pcar** | **−6.49** | **<0.001** |
| Ctai3 – Pphy | −2.63 | 0.152 |
| Ctai4 – Ctai5 | 0.02 | 1.000 |
| Ctai4 – Ctai6 | −2.52 | 0.212 |
| **Ctai4 – Pcar** | **−5.18** | **<0.001** |
| Ctai4 – Pphy | −1.66 | 1.000 |
| Ctai5 – Ctai6 | −2.52 | 0.211 |
| **Ctai5 – Pcar** | **−5.16** | **<0.001** |
| Ctai5 – Pphy | −1.67 | 1.000 |
| Ctai6 – Pcar | −2.66 | 0.140 |
| Ctai6 – Pphy | 0.88 | 1.000 |
| **Pcar – Pphy** | **3.57** | **0.007** |

***Dry weight:***

**The Kruskal–Wallis test revealed a significant effect of the strain on dry weight (χ^2^ = 172.15, df = 8, P < 2.2 × 10^-16^).**

| Comparison | Z statistic | Adjusted P value |
| --- | --- | --- |
| **Csp – Ctai1** | **−11.00** | **<0.001** |
| **Csp – Ctai2** | **−5.44** | **<0.001** |
| **Csp – Ctai3** | **−10.10** | **<0.001** |
| **Csp – Ctai4** | **−10.05** | **<0.001** |
| **Csp – Ctai5** | **−7.69** | **<0.001** |
| **Csp – Ctai6** | **−9.61** | **<0.001** |
| **Csp – Pcar** | **−8.90** | **<0.001** |
| **Csp – Pphy** | **−8.60** | **<0.001** |
| **Ctai1 – Ctai2** | **4.99** | **<0.001** |
| Ctai1 – Ctai3 | 0.96 | 1.000 |
| Ctai1 – Ctai4 | −0.06 | 1.000 |
| Ctai1 – Ctai5 | 2.55 | 0.192 |
| Ctai1 – Ctai6 | 0.47 | 1.000 |
| Ctai1 – Pcar | 1.31 | 1.000 |
| Ctai1 – Pphy | 1.67 | 1.000 |
| **Ctai2 – Ctai3** | **−4.11** | **0.001** |
| **Ctai2 – Ctai4** | **−4.59** | **<0.001** |
| Ctai2 – Ctai5 | −2.22 | 0.473 |
| **Ctai2 – Ctai6** | **−4.12** | **0.001** |
| **Ctai2 – Pcar** | **−3.39** | **0.013** |
| **Ctai2 – Pphy** | **−3.07** | **0.038** |
| Ctai3 – Ctai4 | −0.92 | 1.000 |
| Ctai3 – Ctai5 | 1.69 | 1.000 |
| Ctai3 – Ctai6 | −0.39 | 1.000 |
| Ctai3 – Pcar | 0.44 | 1.000 |
| Ctai3 – Pphy | 0.80 | 1.000 |
| Ctai4 – Ctai5 | 2.38 | 0.315 |
| Ctai4 – Ctai6 | 0.49 | 1.000 |
| Ctai4 – Pcar | 1.24 | 1.000 |
| Ctai4 – Pphy | 1.57 | 1.000 |
| Ctai5 – Ctai6 | −1.90 | 1.000 |
| Ctai5 – Pcar | −1.15 | 1.000 |
| Ctai5 – Pphy | −0.83 | 1.000 |
| Ctai6 – Pcar | 0.75 | 1.000 |
| Ctai6 – Pphy | 1.09 | 1.000 |
| Pcar – Pphy | 0.33 | 1.000 |

***Bacterial load:***

**The Kruskal–Wallis test revealed a significant effect of the strain on bacterial abundance (χ^2^ = 51.106, df = 8, P < 2.504 × 10^-8^ ).**

| Comparison | Z statistic | Adjusted P value |
| --- | --- | --- |
| **Csp – Ctai1** | **−5.74** | **<0.001** |
| **Csp – Ctai2** | **−4.33** | **<0.001** |
| Csp – Ctai3 | −2.45 | 0.259 |
| Csp – Ctai4 | −2.72 | 0.116 |
| Csp – Ctai5 | −2.33 | 0.358 |
| Csp – Ctai6 | −2.58 | 0.179 |
| Csp – Pcar | −0.57 | 1.000 |
| **Csp – Pphy** | **−3.95** | **0.001** |
| Ctai1 – Ctai2 | 1.28 | 1.000 |
| **Ctai1 – Ctai3** | **3.29** | **0.018** |
| **Ctai1 – Ctai4** | **3.01** | **0.047** |
| **Ctai1 – Ctai5** | **3.41** | **0.012** |
| **Ctai1 – Ctai6** | **3.16** | **0.029** |
| **Ctai1 – Pcar** | **5.16** | **<0.001** |
| Ctai1 – Pphy | 1.78 | 1.000 |
| Ctai2 – Ctai3 | 1.94 | 0.944 |
| Ctai2 – Ctai4 | 1.67 | 1.000 |
| Ctai2 – Ctai5 | 2.06 | 0.718 |
| Ctai2 – Ctai6 | 1.81 | 1.000 |
| **Ctai2 – Pcar** | **3.77** | **0.003** |
| Ctai2 – Pphy | 0.47 | 1.000 |
| Ctai3 – Ctai4 | −0.28 | 1.000 |
| Ctai3 – Ctai5 | 0.12 | 1.000 |
| Ctai3 – Ctai6 | −0.13 | 1.000 |
| Ctai3 – Pcar | 1.87 | 1.000 |
| Ctai3 – Pphy | −1.51 | 1.000 |
| Ctai4 – Ctai5 | 0.39 | 1.000 |
| Ctai4 – Ctai6 | 0.14 | 1.000 |
| Ctai4 – Pcar | 2.15 | 0.567 |
| Ctai4 – Pphy | −1.23 | 1.000 |
| Ctai5 – Ctai6 | −0.25 | 1.000 |
| Ctai5 – Pcar | 1.76 | 1.000 |
| Ctai5 – Pphy | −1.62 | 1.000 |
| Ctai6 – Pcar | 2.01 | 0.807 |
| Ctai6 – Pphy | −1.37 | 1.000 |
| **Pcar – Pphy** | **−3.38** | **0.013** |

**SOM section 3: Model estimates of the statistical model fitted to the nodule occupancy data presented in Figure 3A**

A binomial GLMM was fitted to model the **proportion of nodules occupied** by each strain in pairwise co-inoculations (strain1_success), using the competing strain (Sample_abb) as a fixed effect and a random intercept for experiment batch (Expt) (**glmer()**, lme4).

Model: glmer(formula = strain1_success ~ Sample_abb + (1 | Expt), family = binomial(link = "logit"))

Signif. codes: ‘***’ p < 0.001, ‘**’ p < 0.01, ‘*’ p < 0.05

Fixed effect

|  | Estimate | Std. error | z value | Pr(>\|z\|) |  |
| --- | --- | --- | --- | --- | --- |
| (Intercept) | -0.191 | 0.183 | -1.045 | 0.296 |  |
| Ctai1-Ctai3 | 0.592 | 0.244 | 2.422 | 0.015 | * |
| Ctai1-Ctai4 | 0.755 | 0.165 | 4.571 | <0.001 | *** |
| Ctai1-Pcar | -1.182 | 0.231 | -5.122 | <0.001 | *** |
| Ctai2-Csp | 1.179 | 0.303 | 3.888 | <0.001 | *** |
| Ctai2-Ctai5 | -2.792 | 0.386 | -7.231 | <0.001 | *** |
| Ctai3-Ctai5 | 2.894 | 0.361 | 8.016 | <0.001 | *** |
| Ctai3-Pcar | -3.161 | 0.383 | -8.246 | <0.001 | *** |
| Ctai4-Ctai6 | 0.261 | 0.176 | 1.480 | 0.139 |  |
| Pphy-Pcar | 0.056 | 0.215 | 0.260 | 0.795 |  |

Random effect

| Group | Effect | Variance | Std. Dev |
| --- | --- | --- | --- |
| Expt | Intercept | 0.101 | 0.318 |

**SOM section 4: Model estimates of the statistical model fitted to the abundance in single nodule presented in Figure 3B.**

For each strain a GLMM with **gamma distribution** and **log link** was used to compare strain infectivity at the single-nodule level in single vs. co-inoculations (Cp_ml) for each strain, using the sample (either single inoculation or competing strains (Sample_abb)) as a fixed effect and a random intercept for experiment batch (Expt) (**glmer()**, lme4).

Model: glmer(formula = Cp_ml ~ Sample_abb + (1 | Expt), family = Gamma(link = "log"))

Signif. codes: ‘***’ p < 0.001, ‘**’ p < 0.01, ‘*’ p < 0.05

Ctai1:

Fixed effect

|  | Estimate | Std. error | t value | Pr(>\|z\|) |  |
| --- | --- | --- | --- | --- | --- |
| (Intercept) | 16.21260 | 0.20727 | 78.221 | < 0.001 | *** |
| Ctai1-Ctai3 | -0.28737 | 0.24063 | -1.194 | 0.23238 |  |
| Ctai1-Ctai4 | -0.05733 | 0.24321 | - 0.236 | 0.81366 |  |
| Ctai1-Ctai6 | 0.07131 | 0.24451 | 0.292 | 0.77056 |  |
| Ctai1-Pcar | 0.73982 | 0.26010 | 2.844 | 0.00445 | ** |

Random effect

| Group | Effect | Variance | Std. Dev | Levels |
| --- | --- | --- | --- | --- |
| Expt | Intercept | 0.0165 | 0.129 | 7 |
| Residual |  | 0.2689 | 0.519 | 500 obs |

Ctai2:

Fixed effect

|  | Estimate | Std. error | t value | Pr(>\|z\|) |  |
| --- | --- | --- | --- | --- | --- |
| (Intercept) | 16.75300 | 0.09513 | 176.107 | <0.001 | *** |
| Ctai2-Ctai5 | 0.18294 | 0.15996 | 1.144 | 0.253 |  |
| Ctai2-Csp | -0.03961 | 0.10968 | -0.361 | 0.718 |  |

Random effect

| Group | Effect | Variance | Std. Dev | Levels |
| --- | --- | --- | --- | --- |
| Expt | Intercept | 0.001544 | 0.03929 | 4 |
| Residual |  | 0.169593 | 0.41182 | 201 obs |

Ctai3:

Fixed effect

|  | Estimate | Std. error | t value | Pr(>\|z\|) |  |
| --- | --- | --- | --- | --- | --- |
| (Intercept) | 15.72165 | 0.2733 | 57.516 | <0.001 | *** |
| Ctai1-Ctai3 | 0.08834 | 0.31455 | 0.281 | 0.779 |  |
| Ctai3-Ctai5 | -0.01115 | 0.32049 | -0.035 | 0.972 |  |
| Ctai3-Pcar | 0.33157 | 0.34063 | 0.973 | 0.330 |  |

Random effect

| Group | Effect | Variance | Std. Dev | Levels |
| --- | --- | --- | --- | --- |
| Expt | (Intercept) | 0.02194 | 0.1481 | 7 |
| Residual |  | 0.20260 | 0.4501 | 398 obs |

Ctai4:

Fixed effect:

|  | Estimate | Std. error | t value | Pr(>\|z\|) |  |
| --- | --- | --- | --- | --- | --- |
| (Intercept) | 15.8418 | 0.2524 | 62.768 | <0.001 | *** |
| Ctai1-Ctai4 | -0.1110 | 0.2960 | -0.375 | 0.708 |  |
| Ctai4-Ctai6 | 0.1387 | 0.2954 | 0.469 | 0.639 |  |

Random effect

| Group | Effect | Variance | Std. Dev | Levels |
| --- | --- | --- | --- | --- |
| Expt | (Intercept) | 0.01651 | 0.1285 | 4 |
| Residual |  | 0.15435 | 0.3929 | 265 obs |

Ctai5:

Fixed effect

|  | Estimate | Std. error | t value | Pr(>\|z\|) |  |
| --- | --- | --- | --- | --- | --- |
| (Intercept) | 16.0447 | 0.1055 | 152.125 | <0.001 | *** |
| Ctai3-Ctai5 | 0.1235 | 0.1217 | 1.015 | 0.310 |  |
| Ctai5-Csp | 0.1511 | 0.1499 | 1.008 | 0.313 |  |

Random effect

| Group | Effect | Variance | Std. Dev | Levels |
| --- | --- | --- | --- | --- |
| Expt | (Intercept) | 0.003218 | 0.05673 | 4 |
| Residual |  | 0.139341 | 0.37328 | 304 obs |

Ctai6:

Fixed effect

|  | Estimate | Std. error | t value | Pr(>\|z\|) |  |
| --- | --- | --- | --- | --- | --- |
| (Intercept) | 15.7456 | 0.1617 | 97.380 | <0.001 | *** |
| Ctai1-Ctai6 | -0.4122 | 0.1899 | -2.171 | 0.030 | * |
| Ctai4-Ctai6 | -0.1355 | 0.1900 | -0.713 | 0.476 |  |

Random effect

| Group | Effect | Variance | Std. Dev | Levels |
| --- | --- | --- | --- | --- |
| Expt | (Intercept) | 0.01086 | 0.1042 | 4 |
| Residual |  | 0.28803 | 0.5367 | 307 obs |

Pphy:

Fixed effect

|  | Estimate | Std. error | t value | Pr(>\|z\|) |  |
| --- | --- | --- | --- | --- | --- |
| (Intercept) | 16.8678 | 0.6514 | 25.897 | <0.001 | *** |
| Pphy-Pcar | -0.7607 | 0.7494 | -1.015 | 0.31 |  |

Random effect

| Group | Effect | Variance | Std. Dev | Levels |
| --- | --- | --- | --- | --- |
| Expt | (Intercept) | 0.1293 | 0.3596 | 4 |
| Residual |  | 0.2818 | 0.5308 | 169 obs |

Pcar:

Fixed effect

|  | Estimate | Std. error | t value | Pr(>\|z\|) |  |
| --- | --- | --- | --- | --- | --- |
| (Intercept) | 16.2950 | 0.1758 | 92.715 | <0.001 | *** |
| Ctai1-Pcar | -0.1408 | 0.2028 | -0.694 | 0.487617 |  |
| Ctai3-Pcar | -0.1885 | 0.2021 | -0.932 | 0.351187 |  |
| Pphy-Pcar | -0.7578 | 0.2063 | -3.674 | <0.001 | *** |

Random effect

| Group | Effect | Variance | Std. Dev | Levels |
| --- | --- | --- | --- | --- |
| Expt | (Intercept) | 0.01833 | 0.1354 | 4 |
| Residual |  | 0.67097 | 0.8191 | 684 obs |
