# Supplementary material for: Transitive interactions among rhizobia determine their symbiotic fitness
Source: ISME Commun. 2026 Jul 21;6(1):ycag210. doi: 10.1093/ismeco/ycag210 (PMC13557124; doi:10.1093/ismeco/ycag210)
Supplement: Supplementary file 1 [file supplementary_material_ycag210.zip › Supplementary_figures_SELECT_alt_text_ycag210.pdf]

Supplementary figures for the manuscript:

Transitive interactions among rhizobia determine bacterial fitness during their interaction with the legume plant *Mimosa pudica*

Authors:

Margarita Granada Agudelo, Bryan Ruiz, Jean-Baptiste Ferdy, Delphine Capela, Philippe Remigi

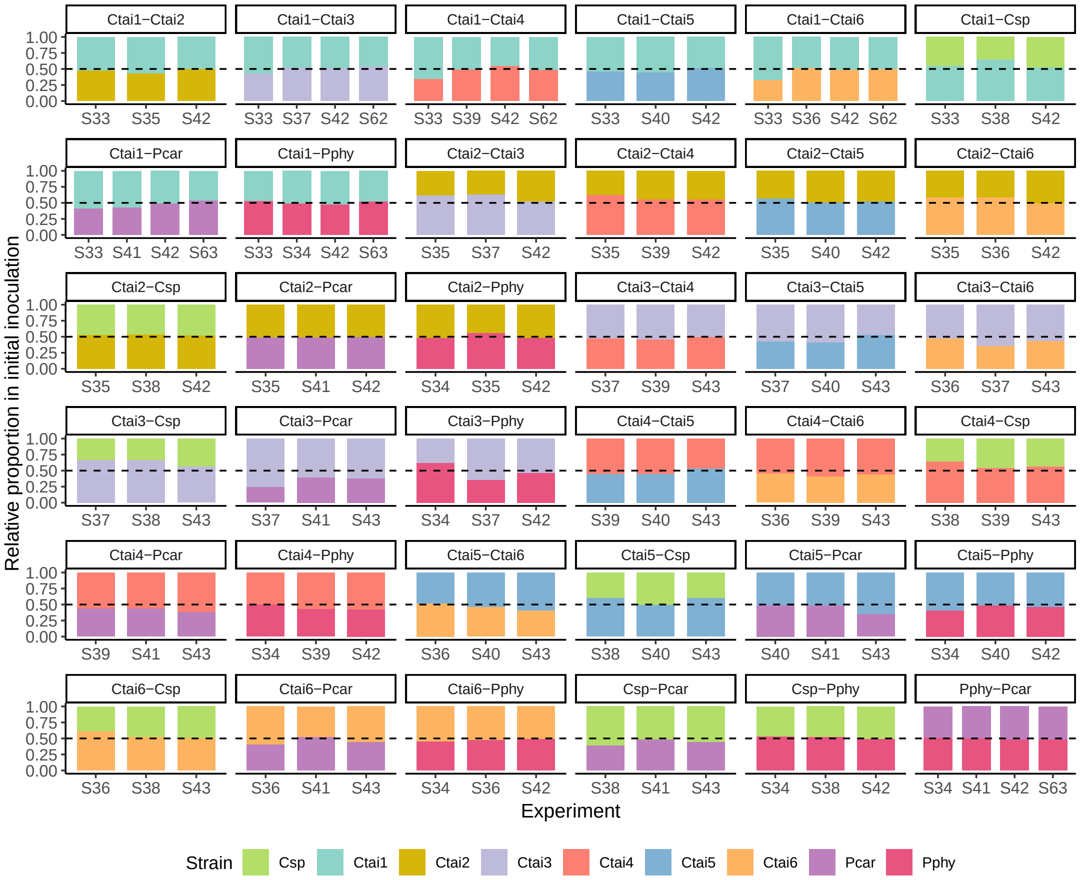

Supplementary Figure 1. Initial proportions of each strain in co-inoculation experiments. Experiments were assigned unique identifiers (S33 to S63, with gaps), displayed on the x-axis.

ALT TEXT: A grid of 36 sub-panels containing stacked bar plots showing the relative proportions of each strain at inoculation in co-inoculation experiments. Each panel represents a pair of strains, with experiments identified by unique labels ranging from S33 to S63. Most panels show approximately equal starting proportions across experiments, though moderate variation is visible in some pairs.

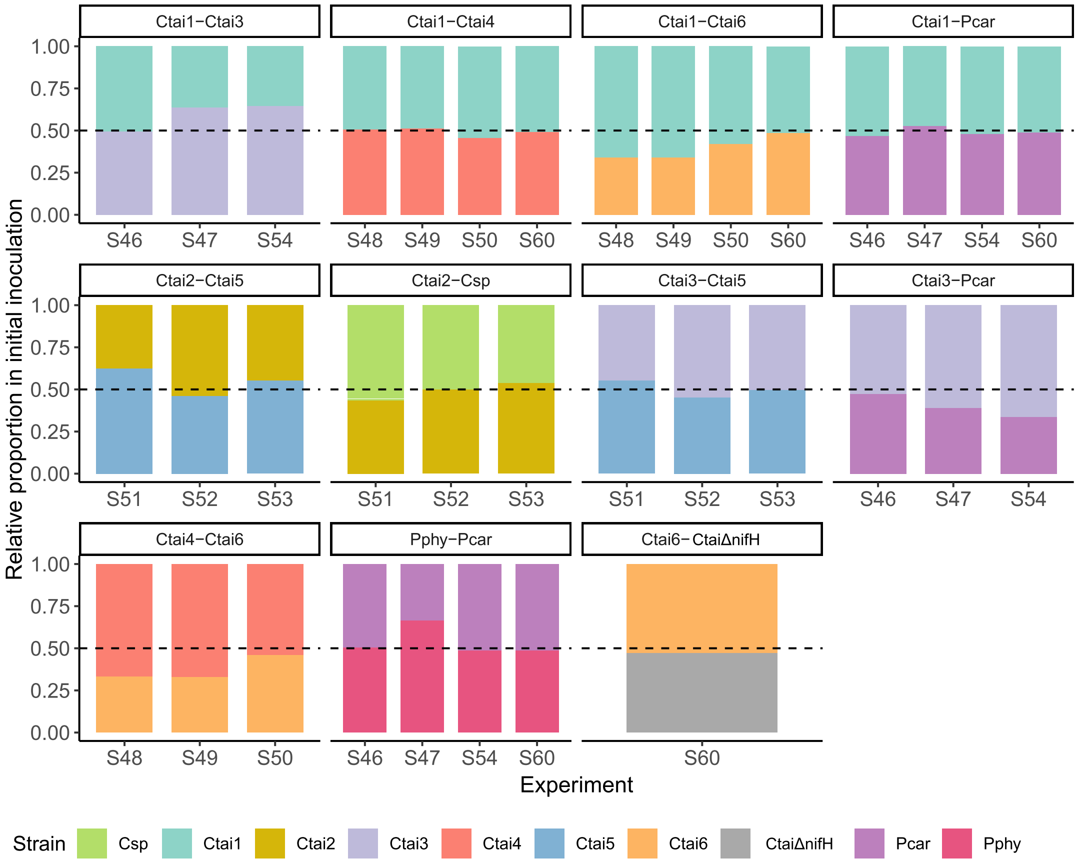

Supplementary Figure 2. Initial proportions of each strain in nodulation competitiveness experiments. Experiments were assigned unique identifiers (S46 to S60, with gaps), displayed on the x-axis.

ALT TEXT: A grid of 12 sub-panels containing stacked bar plots showing the relative proportions of each strain at inoculation in nodulation competitiveness experiments. Each panel represents a pair of strains, with experiments identified by unique labels ranging from S46 to S60. Most panels show approximately equal starting proportions across experiments, though moderate variation is visible in some pairs.

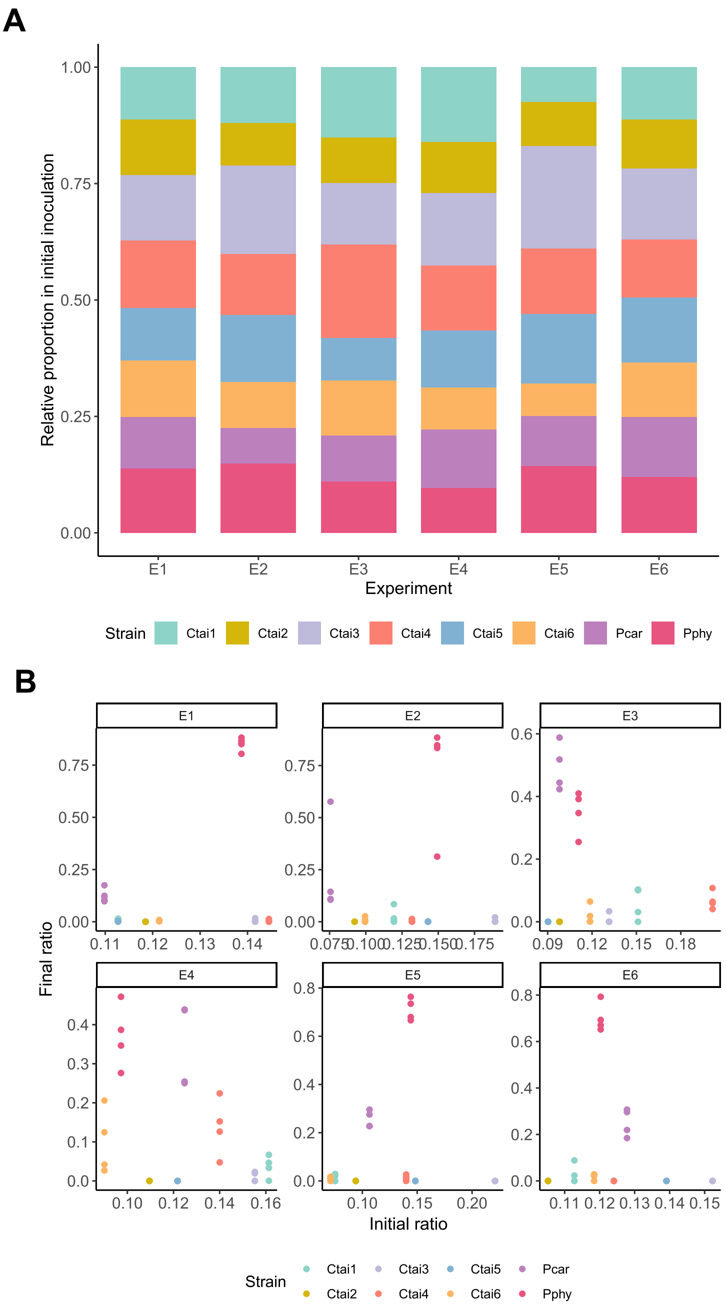

Supplementary Figure 3. (A) Initial proportions of each strain in the 8-strain community inoculation experiments. **(B)** Correlation between initial (inoculum) and final (in nodules) proportion of each strain. Each point represents one technical replicate in each experiment.

ALT TEXT: Two-panel figure (A and B) showing strain proportions at inoculation and their relationship to final nodule occupancy in the 8-strain community inoculation experiments. Panel A is a stacked bar plot showing the relative proportion of each of the 8 strains at initial inoculation for each experiment. Panel B shows six scatter plots displaying the correlation between the initial proportion of each strain in the inoculum and its final proportion in nodules.

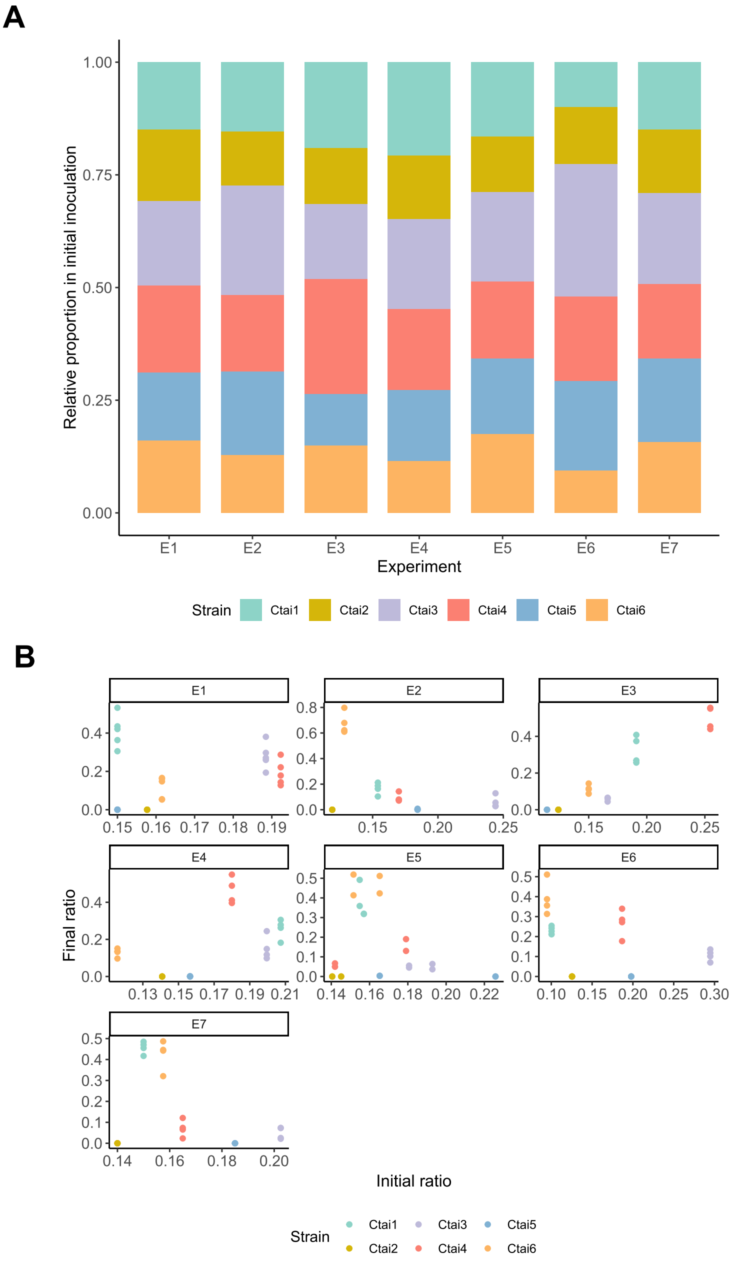

Supplementary Figure 4. **(A)** Initial proportions of each strain in the 6-strain community inoculation experiments. **(B)** Correlation between initial (inoculum) and final (in nodules) proportion of each strain. Each point represents one technical replicate in each experiment.

ALT TEXT: Two-panel figure (A and B) showing strain proportions at inoculation and their relationship to final nodule occupancy in the 6-strain community inoculation experiments. Panel A is a stacked bar plot showing the relative proportion of each of the 6 strains at initial inoculation for each experiment. Panel B shows six scatter plots displaying the correlation between the initial proportion of each strain in the inoculum and its final proportion in nodules.

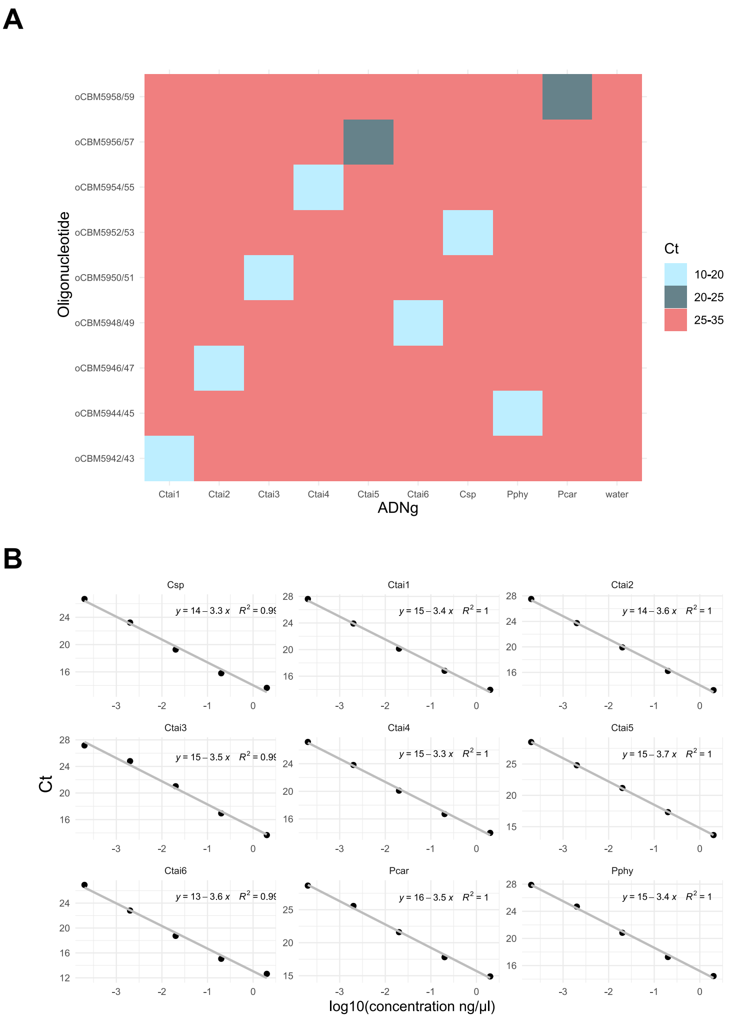

Supplementary Figure 5. Validation of qPCR oligonucleotides used in this study. **(A)** Specificity of qPCR oligonucleotides tested against genomic DNA preparations of all rhizobial strains. Ct of each qPCR reaction is indicated in colored bins. **(B)** Efficiency of qPCR oligonucleotides tested against genomic DNA dilutions of their respective target strains. Equations and R^2 values of linear fits of Ct values against logarithmic values of DNA concentrations are shown on each plot. The qPCR efficiency was calculated from the slope of each standard curve. Efficiencies were as follows: Csp = 100%, Ctai1 = 97%, Ctai2 = 90%, Ctai3 = 93%, Ctai4 = 100%, Ctai5 = 86%, Ctai6 = 90%, Phy = 93%, and Pcar = 97%.

ALT TEXT: Two-panel figure (A and B) showing the specificity and efficiency of qPCR oligonucleotides used in this study. Panel A is a heatmap showing the Ct values obtained when each oligonucleotide pair was tested against genomic DNA from all rhizobial strains and water. Panel B shows 9 standard curves, one per oligonucleotide pair. Each plot includes a linear fit with the corresponding equation and R2 value.

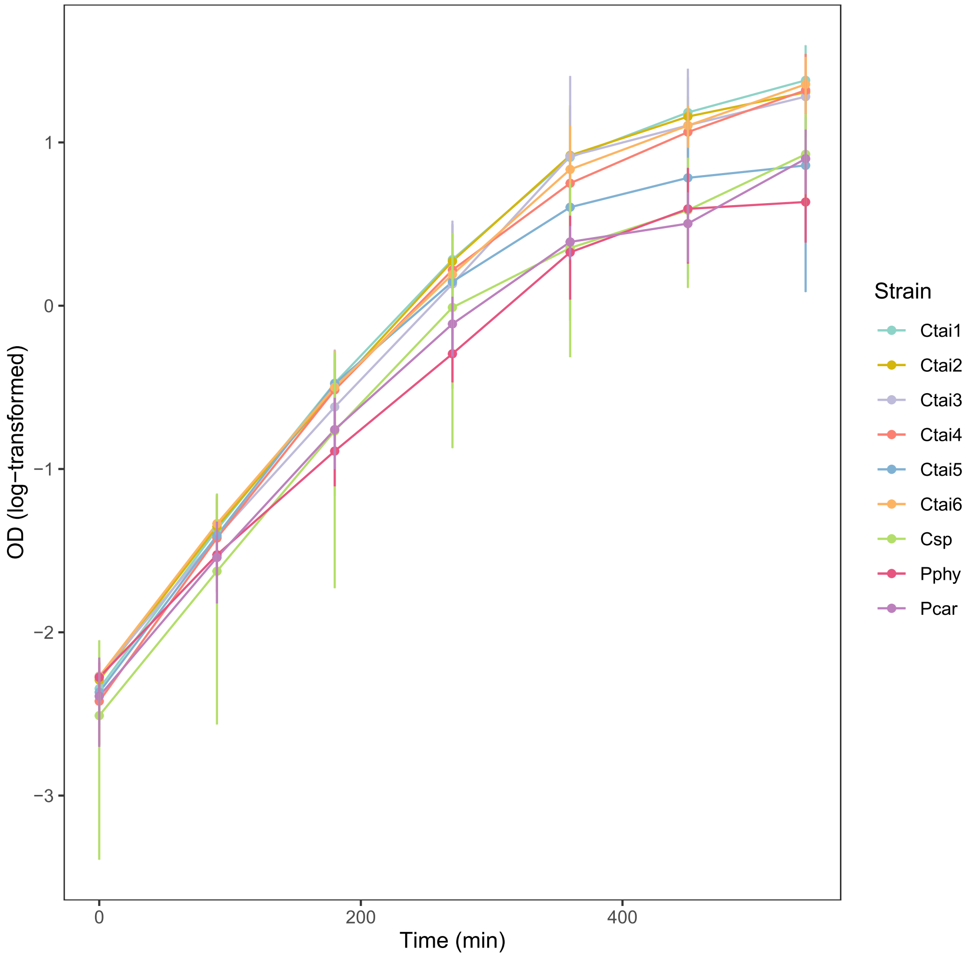

Supplementary Figure 6. Growth curves of the 9 rhizobial strains in TY medium. Points indicate the mean of four biological replicates, and error bars represent the standard deviation.

ALT TEXT: A line graph showing the growth curves of the 9 strains over time in TY rich medium. All strains show a similar growth curve.

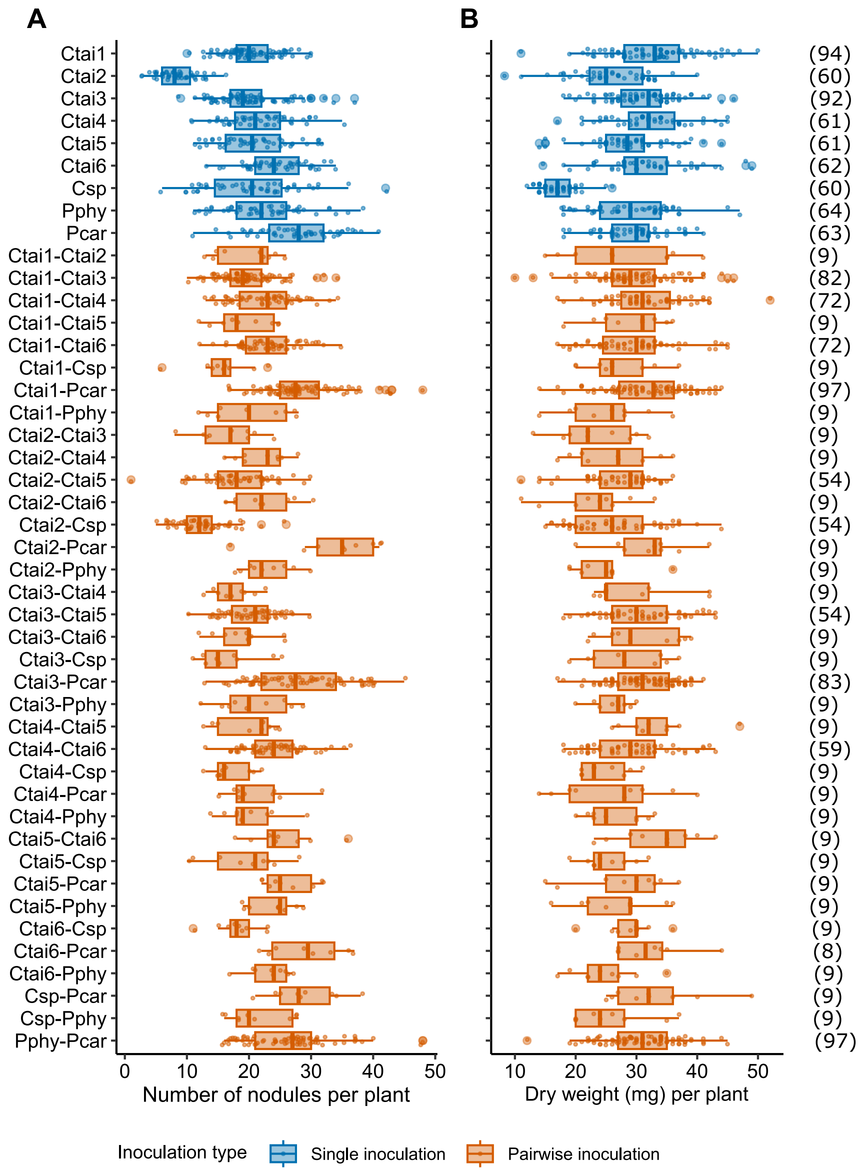

Supplementary Figure 7. Comparison of symbiotic phenotypes in single and pairwise inoculations. **(A)** Number of nodules per plant. **(B)** Shoot dry weight measured in *M. pudica* plants inoculated with the different bacterial strains and all their pairwise combinations. Measurements were performed at 28 dpi and come from all the independent experiments of this study. Sample sizes are indicated between brackets.

ALT TEXT: Two-panel figure (A and B) comparing symbiotic phenotypes between single and pairwise inoculations across 9 strains and their 36 pairwise combinations. Panel A shows the number of nodules per plant and Panel B shows shoot dry weight in mg per plant. In both panels, horizontal blue boxplots represent single-inoculation and orange boxplots represent pairwise co-inoculations, with individual data points overlaid.

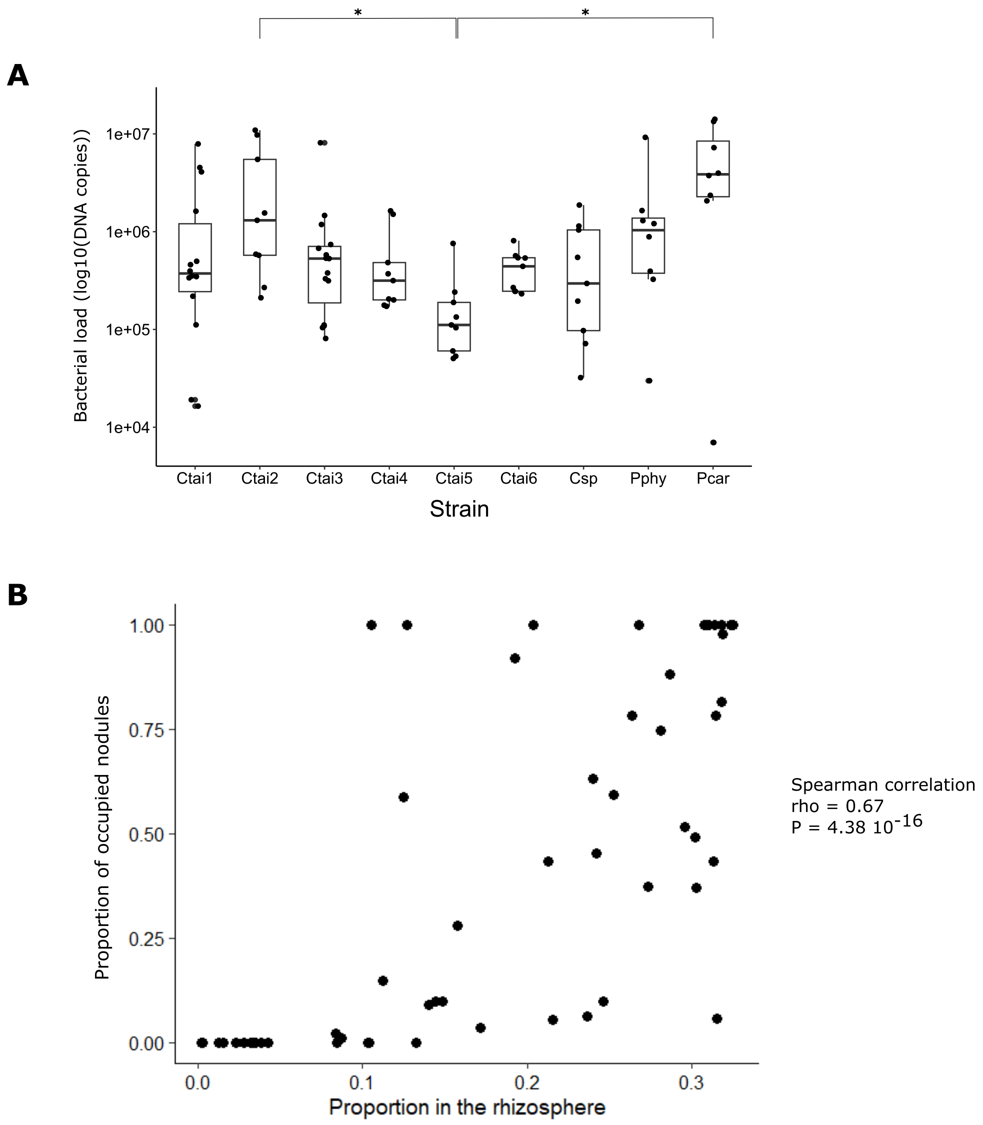

Supplementary Figure 8. Rhizosphere colonisation. **(A)** Bacterial abundance in the rhizosphere during single inoculations. n $\geq$ 9. Kruskal–Wallis rank sum test with Dunn's post-hoc correction. Asterisks indicate p-values < 0.05. **(B)** Correlation between relative bacterial abundance in the rhizosphere and nodulation occupancy during pairwise inoculations. Each point represents, for one focal strain of a given pair, the mean values of rhizosphere proportions and nodulation competitiveness measured in one experiment. n = 3.

ALT TEXT: Two-panel figure (A and B) showing rhizosphere colonisation and its relationship to nodule occupancy. Panel A is a box plot showing bacterial abundance in the rhizosphere for each of the 9 strains during single inoculations, with individual data points overlaid. Panel B is a scatter plot showing the correlation between the relative proportion of each strain in the rhizosphere and its proportion of occupied nodules during pairwise inoculations.

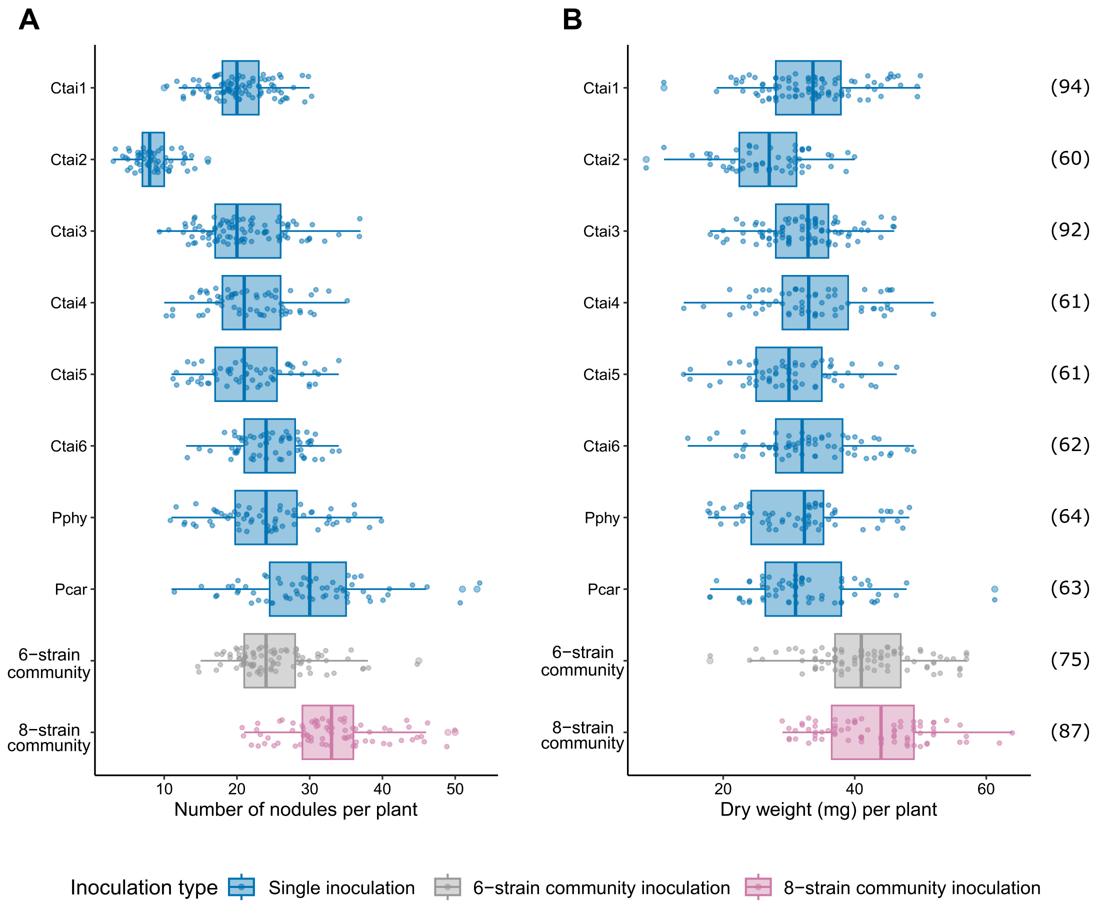

Supplementary Figure 9. Comparison of symbiotic phenotypes in single and community inoculations. **(A)** Number of nodules per plant. **(B)** Shoot dry weight measured in *M. pudica* plants inoculated with the different bacterial strains and with the 6-strain and 8-strain communities. Measurements were performed at 28 dpi and come from all the independent experiments of this study. Sample sizes are indicated between brackets.

ALT TEXT: Two-panel figure (A and B) comparing symbiotic phenotypes between single and community inoculations across 8 strains and a 6-strain or 8-strain communities. Panel A shows the number of nodules per plant and Panel B shows shoot dry weight in mg per plant. In both panels, horizontal blue boxplots represent single-inoculation, light-grey boxplots represent the 6-strain community and light-purple boxplots represent the 8-strain community, with individual data points overlaid.

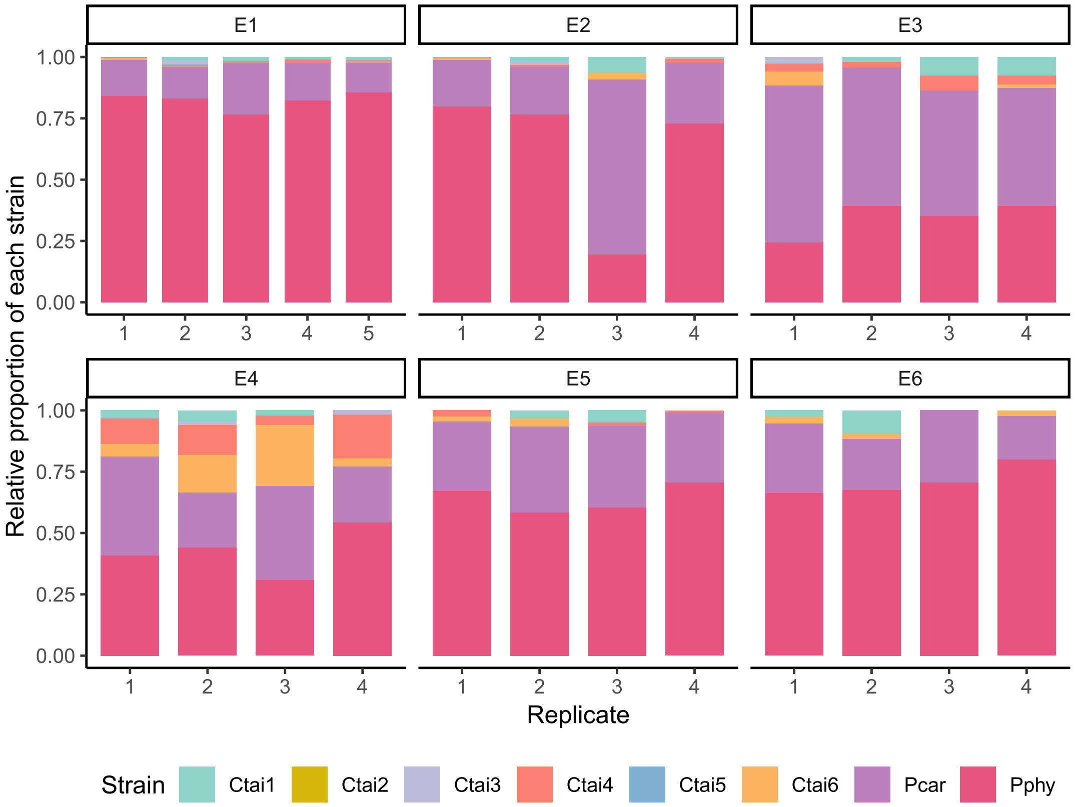

Supplementary Figure 10. Relative bacterial abundances in individual replicates from 6 independent experiments (E1-E6) inoculated with the 8-strain community. Each experiment comprised 4 or 5 replicate groups of 3 plants from which nodule bacterial populations were extracted and quantified by qPCR.

ALT TEXT: A grid of six stacked bar plots, one per independent experiment, showing the relative proportion of each strain in nodule bacterial populations across individual replicates in the 8-strain community inoculations. The relative proportion ranges from 0.00 to 1.00, and the replicate number ranges between 4 and 5 depending on the experiment. Pphy and Pcar dominate nodule occupancy across most replicates and experiments, with other strains having smaller and more variable proportions.

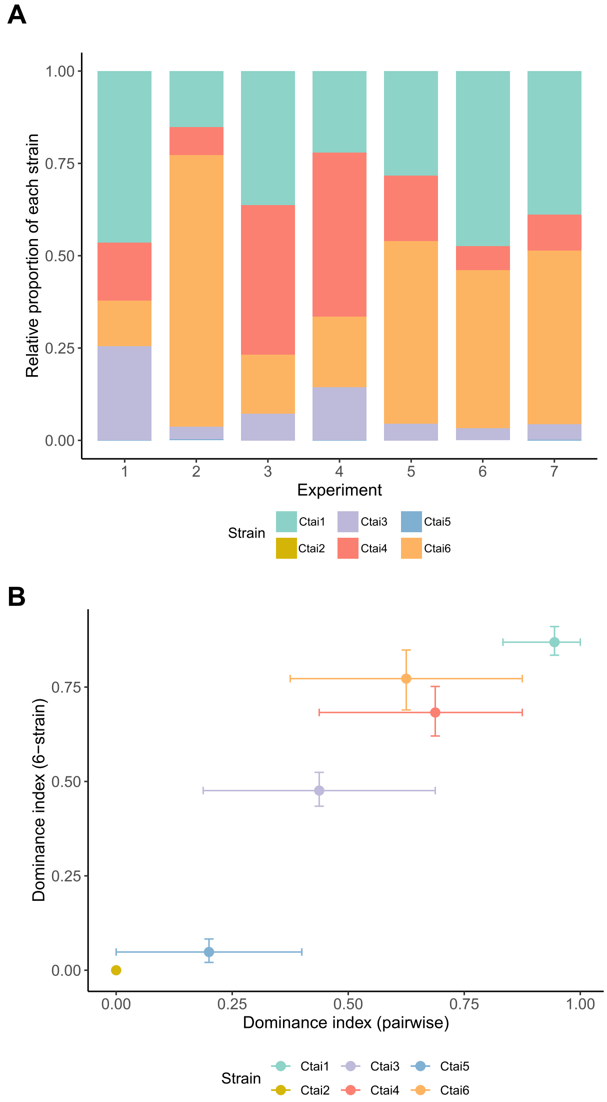

Supplementary Figure 11. Symbiotic fitness in the *C. taiwanensis* 6-strain community. **(A)** Mean relative frequencies of strain abundances in the 6-strain community. Data from 7 independent experiments are shown, each comprising 4 or 5 replicate groups of 3 plants. **(B)** Dominance index from the 6-strain community plotted against dominance index from pairwise inoculations.

ALT TEXT: Two-panel figure (A and B) showing symbiotic fitness of the six C. taiwanensis strains in community inoculations. Panel A consists of stacked bar plots showing mean strain relative abundances in 6-strain communities. Panel B is a scatter plot comparing dominance indices from pairwise inoculations and 6-strain communities. Each point represents the mean dominance index of one strain, and error bars indicate variability around each mean.

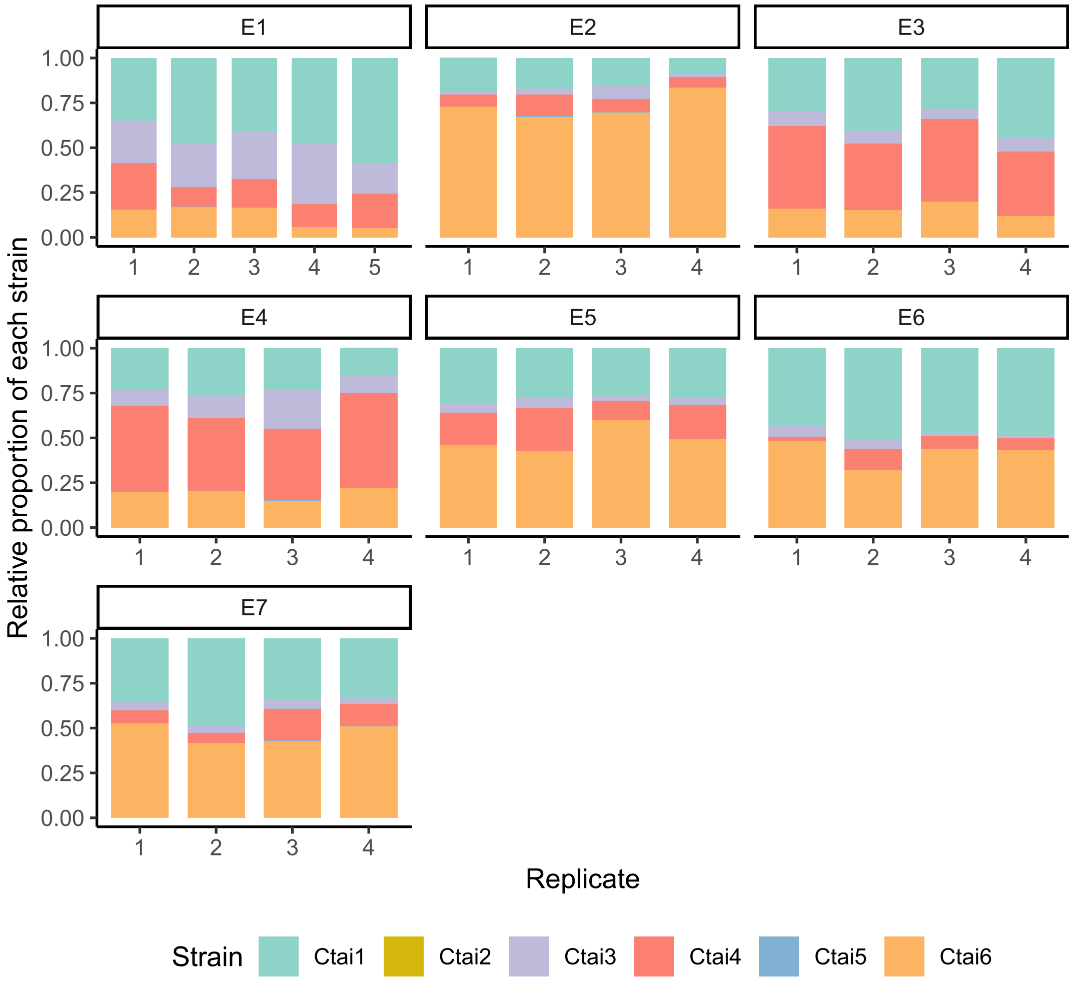

Supplementary Figure 12. Relative bacterial abundances in individual replicates from 7 independent experiments (E1-E6) inoculated with the 6-strain community. Each experiment comprised 4 or 5 replicate groups of 3 plants from which nodule bacterial populations were extracted and quantified by qPCR.

ALT TEXT: A grid of seven stacked bar plots, showing the relative proportion of each strain in nodule bacterial populations in individual replicate groups in the 6-strain community inoculations. Strain dominance varies across experiments but remains consistent among replicates within the same experiment.
